# Insights into subspecies classification and conservation priorities of Central Asian lynx populations revealed by morphometric and genetic analyses

**DOI:** 10.1038/s41598-024-55807-x

**Published:** 2024-03-02

**Authors:** Nazerke Bizhanova, Olga Nanova, Davoud Fadakar, Alexey Grachev, Zijia Hong, Shahrul Anuar Mohd Sah, Zhansaya Bizhanova, Mikhail Sablin, Yuriy Grachev

**Affiliations:** 1 Laboratory of Theriology, Institute of Zoology, 050060 Almaty, Kazakhstan; 2 Wildlife Without Borders Public Fund, 050063 Almaty, Kazakhstan; 3 Zoological Museum, M.V. Lomonosov Moscow State University, 119991 Moscow, Russia; 4 Department of Natural Resources, Isfahan University of Technology, Isfahan, 84156‑83111 Iran; 5 School of Biological Sciences, Universiti Sains Malaysia, 11800 USM Penang, Malaysia; 6 School of Medicine, Nazarbayev University, 020000 Astana, Kazakhstan; 7 Zoological Institute, Russian Academy of Sciences, 199034 Saint Petersburg, Russia

**Keywords:** Phylogenetics, Haplotypes, Taxonomy, Adaptive radiation, Evolutionary ecology

## Abstract

The Eurasian lynx *(Lynx lynx*) exhibits geographic variability and phylogenetic intraspecific relationships. Previous morphological studies have suggested the existence of multiple lynx subspecies, but recent genetic research has questioned this classification, particularly in Central Asia. In this study, we aimed to analyse the geographic and genetic variation in Central Asian lynx populations, particularly the Turkestan lynx and Altai lynx populations, using morphometric data and mtDNA sequences to contribute to their taxonomic classification. The comparative analysis of morphometric data revealed limited clinal variability between lynx samples from the Altai and Tien Shan regions. By examining mtDNA fragments (control region and cytochrome *b*) obtained from Kazakhstani lynx populations, two subspecies were identified: *L. l. isabellinus* (represented by a unique haplotype of the South clade, H46) and *L. l. wrangeli* (represented by haplotypes H36, H45, and H47 of the East clade). *L. l. isabellinus* was recognized only in Tien Shan Mountain, while Altai lynx was likely identical to *L. l. wrangeli* and found in northern Kazakhstan, Altai Mountain, Saur and Tarbagatai Mountains, and Tien Shan Mountain. The morphological and mtDNA evidence presented in this study, although limited in sample size and number of genetic markers, renders the differentiation of the two subspecies challenging. Further sampling and compilation of whole-genome sequencing data are necessary to confirm whether the proposed subspecies warrant taxonomic standing.

## Introduction

The genetic differentiation and phylogeographic structure for subspecies formation in mammalian carnivores of the Northern Hemisphere has been shaped by historical climatic and geological events, in addition to contemporary human-driven impacts such as habitat destruction, fragmentation, and modification^1,2^. The conservation and classification of modern large carnivores present significant challenges due to their widespread distribution, high mobility, and susceptibility to human activities^3^. Among these carnivores, the phylogeography of the Eurasian lynx (*Lynx lynx*) is of great interest, as this species represents a suitable model to explore geographic variability due to its extensive distribution across diverse landscapes and significant morphological variation and specialization in predation^4^.

The subspecies delimitation of the Eurasian lynx has historically relied on morphological studies, which compared variables such as cranial characteristics, body size, colour and spotting of skins, distribution patterns of alleged subspecies, and the presence of geographical barriers (i.e., Heptner & Sludskiy^5^, Wozencraft^6^, Kitchener et al.^7^). The mentioned studies recognised five to ten subspecies. However, with the introduction of molecular genetic techniques in systematic and phylogeographic studies, the number of proposed subspecies has significantly decreased, identifying three to five major clades of lynx populations^8–10^. The existence of subspecies has been called into question, suggesting that the morphological variability that supported the previous subspecies distinctions may be a plastic response to local environmental changes^9^. Despite the discrepancy between the results of phylogeographic research and the subspecies concept according to the biological species concept, using the subspecies concept for conservation and current taxonomic purposes may be justified.

Previous studies based on mitochondrial DNA (mtDNA) sequences and microsatellite markers have recognised the northern lynx (*L. l. lynx*), Turkestan or Himalayan lynx (*L. l. isabellinus*), and Siberian lynx (*L. l. wrangeli*) as the most reliable subspecies, followed by the Caucasian lynx (*L. l. dinniki*)^3^, while the subspecies status of the Balkan (*L. l. balcanicus*), Carpathian (*L. l. carpathicus*), Baikal (*L. l. kozlovi*), and Altai lynxes (*L. l. wardi*) require further confirmation or refutation^3,5,11,12^. Behzadi et al.^10^ found four clades in the phylogenetic analysis: South, East, West 1 and West 2, based on the mitochondrial control region (CR) sequences. By comparing these clades with other CR clades^8^ and whole mitochondrial genome clades^9^, three subspecies were proposed, namely, the Siberian lynx (*L. l. wrangeli*) corresponding to the East clade, the northern lynx (*L. l. lynx*), including two mitochondrial clades, i.e., clade West 1 and West 2, and all southern localities, including populations in the Himalaya region, Tibet, Iran, Iraq, Turkey, and the Caucasus region, correspond with the South clade considered as *L. l. isabellinus*.

The genetic structure of the species has been well studied in Europe, where limited genetic variation and pronounced differentiation within northern lynx populations have been found, along with considerable gene flow across the subspecies’ central range^13–15^. Gugolz et al.^16^ proposed a scenario of postglacial recolonization of the northern lynx within Europe after the Last Glacial Maximum (LGM). Rueness et al.^8^ utilized mitochondrial CR and cytochrome *b* (cyt *b*) sequences, along with microsatellites, to reveal that the oldest lynx lineage can be traced back to Central Asia, suggesting that the origins of Eurasian lynx populations can be attributed to Central Asia, with subsequent expansion towards northwestern Siberia and Scandinavia following the LGM.

In contrast to European populations, the range of the species in Asia has been characterised as uninterrupted, with thriving and interconnected populations^8–10^. However, few genetic studies have been conducted for Asian lynx populations^17,18^, and one open question in intraspecific taxonomy pertains to the Central Asian lynx populations and the potential subspecies inhabiting this region. There has long been debate over the subspecies status of the Turkestan lynx and Altai lynx in the northern part of Central Asia, mostly in Kazakhstan, where there might be a border between these two taxa^7^. While there has not been a comprehensive comparative analysis on differentiation between these lynx populations, there is an assumption about the possibility of the Altai lynx being identical to or a variation of the Turkestan lynx^5,19^. The Altai lynx was initially described as a subspecies by Lydekker^20^, who highlighted its distinct features, such as its light colouration and specific markings. However, Satunin^21^ argued that the Altai lynx’s characteristics were similar to those of the northern lynx, leading to its classification as such. Ognev’s^19^ examination of lynx skins from Altai revealed similarities in colouration with the Turkestan lynx described in Tibet, causing the systematic position of the Altai lynx to be uncertain. Conversely, Stroganov^22^, based on his study of lynx skulls (*n* = 19) and skins (*n* = 10) from the Russian Altai, strongly advocated for classifying the Altai lynx as a separate subspecies. The author highlighted unique characteristics such as pronounced flattening of the skull’s frontal bone and a relatively large size, placing the Altai lynx between the smaller northern lynx and the larger Siberian lynx. Sludskiy^23^ also initially classified the Altai lynx as the northern lynx, but subsequent collaborations with Heptner^5^ and further research indicated a potential close relationship between the Altai and Turkestan lynxes. This prompted a re-evaluation, with Sludskiy^24^ excluding the Altai lynx from the list of subspecies and calling for additional research to clarify its classification.

As the Turkestan lynx is rare or endangered throughout almost all countries of its habitat^25,26^, compared to the more abundant Altai lynx^27^, research on defining these two taxa is crucial for reconsidering or reinforcing the conservation status of the Turkestan lynx and addressing the intriguing taxonomic question. The taxonomic position of the lynx inhabiting the Saur and Tarbagatai Mountains near the Irtysh River (a possible barrier between Turkestan lynx and Altai lynx^7^), located between the Altai Mountains region and Tien Shan-Zhetisu Alatau Mountains region in the northern part of Central Asia, has also never been properly classified to any of these subspecies^28^.

In this paper, we analysed the geographic variation of the Eurasian lynx in Central Asia using morphometric data from osseous specimens and analysed its genetic variation across most of the species’ geographical range using mtDNA sequences. Specifically, we examined the phylogeographic structure of the Central Asian lynx populations to determine whether subspecies recognition of Altai lynx and Turkestan lynx is warranted. The results might help us gain insight into the taxonomic position of the lynxes studied, formulate conservation strategies and ensure sustainable management of lynx populations in Asia.

## Results

### Cranial characteristics

At baseline, the effects of AGE (*F* = *588.4, p* = *0.033, df* = *26*), SEX (*F* = *8.5, p* = *0.027, df* = *78*), and LOC (*F* = *10.5, p* < *0.001, df* = *108*) were significant on log-transformed data. On Burnaby-corrected data, the effects of AGE (*F* = *9.0, p* = *0.105, df* = *25*) and SEX (*F* = *1.7, p* = *0.227, df* = *75*) were not statistically significant, and the effect of LOC was reliable (*F* = *18.4, p* = *0.001, df* = *108*). We conducted a further study of geographic variability on data modified using Burnaby’s procedure, from which age and sex variations were excluded. This procedure enabled us to employ all measured specimens as a unified sample for geographic variability analysis.

**Figure 1:**
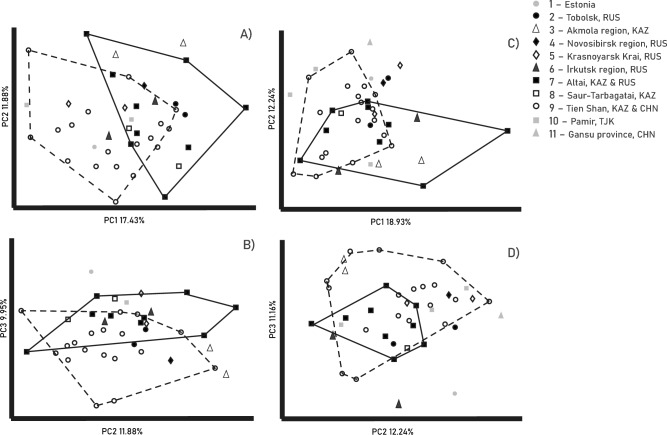
Plot of the Eurasian lynx (*Lynx lynx*) specimen on the space of the first three principal components (PC): (**A**) cranium, PC1 vs. PC2, (**B**) cranium PC2 vs. PC3, (**C**) mandible, PC1 vs. PC2, (**D**) mandible PC2 vs. PC3. Data adjusted for sex and age variability. The solid line outlines the sample from Altai, while the dotted line outlines the sample from Northern Tien Shan. Specimens come from Kazakhstan (KAZ), Russia (RUS), China (CHN), Tajikistan (TJK) and Estonia.

For the principal component analysis, a total of 25 major components were identified for the cranium. The cranium variability is poorly structured (Supplementary Fig. S1a). The first principal component, PC1, explained 17.43% of the total variability. The second principal component (PC2) accounted for 11.88%, and the third component (PC3) for 9.95%. The proportion of variability gradually decreased for the subsequent principal component. Samples from Altai (number 7) and Tien Shan (number 9) were partly separated by PC1 and to a lesser extent by PC3 (Fig. 1a,b). The sample from Saur-Tarbagatai (number 8) was located in the area where samples from Altai and Tien Shan overlapped in the PC1-PC2 and PC2-PC3 spaces. In the remaining principal components, which represent a smaller proportion of variability, the samples overlapped. The loadings of the three major components for the cranium can be found in Supplementary Tables S5, S6.

**Figure 2:**
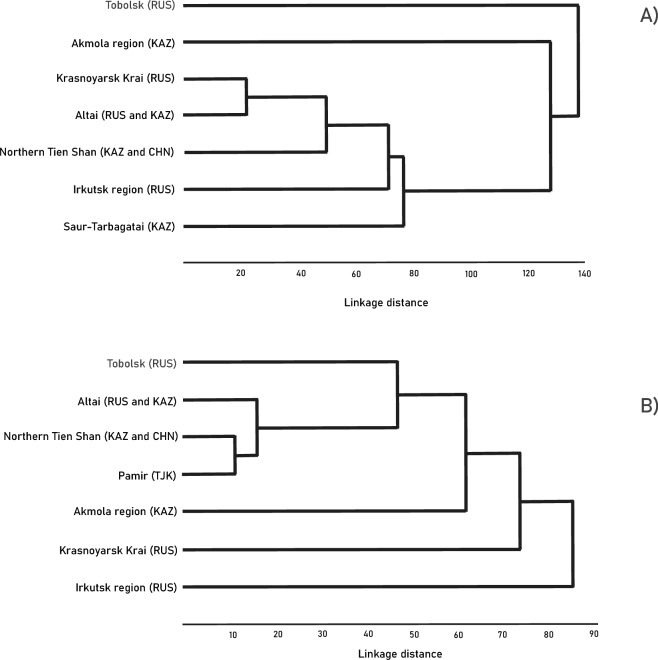
Branching tree diagram resulting from the morphological congruence analysis based on morphological characteristics: (**A**) 27 cranial characteristics; (**B**) 21 mandibular characteristics. UPGMA method, Mahalanobis quadratic distances. Specimens come from Kazakhstan (KAZ), Russia (RUS) and China (CHN).

Based on the cluster analysis, the dendrogram in Fig. 2a illustrates that the sample from Altai (number 7) was classified as closest to the sample from the Krasnoyarsk Territory (number 5). The sample from Tien Shan (number 9) was located next to this group. The dendrogram was constructed using the Mahalanobis quadratic distances matrix with their reliability checked, which can be found in Supplementary Tables S7–S10.

A pairwise comparison of the means for samples 7 and 9 for each of the 27 corrected features using the Bonferroni correction (a Bonferroni-corrected Mann–Whitney U-test) (*p* < *0.001852*) revealed that these samples differed in one feature, specifically the zygomatic width. For more details, see Supplementary Table S11.

To assess whether there were variations in cranial dimensions from the northern to southern regions, we calculated correlations between the condylobasal length of the cranium and the latitude of specimen capture. Calculations were carried out on logarithmic data; only adult animals were included in the analysis, and correlations were calculated separately for males and females. The results indicated that there was no correlation between condylobasal cranial length and breadth with latitudinal location for either males (r = 0.3, p = 0.34, n = 12) or females (r = -0.07, p = 0.86, n = 9).

### Mandibular characteristics

The log-transformed data analysis showed that the effect of age (AGE, *F* = *2.6, p* = *0.025, df* = *22*) and locality (LOC, *F* = *34.0, p* = *0.020, df* = *80*) on the study were found to be significant, while the effects of sex (SEX, *F* = *1.6, p* = *0.051, df* = *66*) was close to being significant. The effect of LOC was significant. In contrast, when corrected using Burnaby’s procedure, the effects of age (*F* = *0.32, p* = *0.99, df* = *19*), sex (*F* = *0.79, p* = *0.81, df* = *57*), and locality (*F* = *30.60, p* = *0.1, df* = *54*) were not significant.

We conducted further analyses of geographic variability on data modified using Burnaby’s procedure, from which age and sex variations were excluded. This enabled us to combine sex and age groups into a single sample.

In the principal component analysis for the mandible, a total of 19 major components were identified. The first principal component, PC1, explained 18.93% of the total variability, while the second principal component, PC2, accounted for 12.24%, and PC3 explained 11.16% (Supplementary Fig. S1b). As seen in Fig. 1c,d, the variability was not well structured. Samples from Altai (number 7) and Tien Shan (number 9) were partially separated by the PC1 but completely overlapped in the PC2-PC3 space. The Saur-Tarbagatai sample (number 8) was in the area where the samples from Altai and Tien Shan overlapped in the PC1-PC2 space (as shown in Fig. 1c,d). The loadings of the major components for the mandible are presented in Supplementary Table S6.

The dendrogram (Fig. 2b) from the cluster analysis illustrates that the sample from Tien Shan (number 9) clustered with the sample from Pamir and Tajikistan (number 10). Additionally, the sample from Altai (number 7) was sister to this group. We created the dendrogram using the Mahalanobis quadratic distances and checked their reliability, as presented in Supplementary Tables S9 and S10, respectively.

Pairwise comparison of means of the Altai and Northern Tien Shan samples for each of the 21 corrected traits using the Bonferroni correction (a Bonferroni-corrected Mann–Whitney U-test) (*p* < *0.002381*) revealed that there were no differences in any of the mandible traits analysed. For more details, see Supplementary Table S12.

We calculated correlations between the length of the mandible and the latitude of the specimen capture to assess whether there was dimensional variability in the samples from north to south. The analysis included only adult specimens, and the correlations were calculated separately for males and females using logarithmic data. The results showed no correlation between the mandible length and width with latitudinal location for either males (r = − 0.35, p = 0.26, n = 12) or females (r = − 0.36, p = 0.31, n = 10).

Table 1 presents the frequency of dental features for samples from Altai and Northern Tien Shan. Morphotype 2 was more frequent in specimens from Tien Shan, while morphotype 3, M_1_ with a metaconid, was more frequent in the specimens from Altai. Molar with no mesiolingual cusp or inflection was more frequent for the samples from the Tien Shan area.

**Table 1:** Frequency of morphotypes for Eurasian lynx (*Lynx lynx*) samples from Altai and Northern Tien Shan.

Frequency	Morphotype 1	Morphotype 2	Morphotype 3	Total, %
Altai, n = 9	11.1	33.3	55.6	100
Northern Tien Shan, n = 18	16.7	50.0	33.3	100

### Phylogenetic analysis

In total, the control region (CR) fragment of the seven samples of Eurasian lynx from Kazakhstan (three samples from the Northern Tien Shan Mountains, two samples from the Saur-Tarbagatai Mountains, one from the Altai Mountains, and one from the Akmola region) was successfully amplified. All new mtDNA sequences were submitted to GenBank (accession numbers OR837122-OR837135) (Supplementary Table S4). These sequences were aligned with 85 published Eurasian lynx CR sequences retrieved from GenBank NCBI (https://www.ncbi.nlm.nih.gov/genbank/). The available mtDNA sequences were slightly trimmed to match our sequence length, so we added only 38 instead of the 48 haplotypes detected by Rueness et al.^8^, as well as six haplotypes from other sources^10,14,15^. See Supplementary Table S4 for the list of mtDNA sequences and corresponding haplotype information.

**Figure 3:**
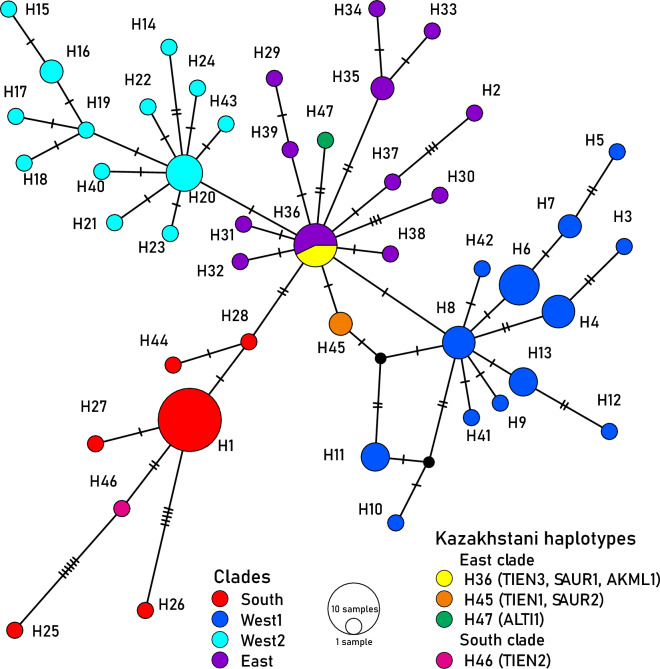
Median-joining network based on the CR gene depicting the relationships between the major four groups described for Eurasian lynx, including East (purple), South (red), West 1 (blue), and West 2 (turquoise). The colours correspond to the respective clades in the phylogenetic tree. Additional details of haplotypes and accession numbers are provided in Supplementary Table S4, supporting information. Black small dots represent missing haplotypes, circle sizes are proportional to haplotype frequencies, and numbers are haplotype numbers. The colours of Kazakhstani haplotypes (H36, H45, H46, and H47) are shown in the legend.

We followed Behzadi et al.^10^ for the infraspecific classification of *L. lynx* [proposing three subspecies *L. l. wrangeli* (East clade), *L. l. lynx* (West 1 and West 2), and *L. l. isabellinus* (South clade) by comparing CR and the whole mitochondrial genome clades] along with haplotype numbers and relative clades of CR. Based on 613 bp of CR from 92 mtDNA sequences, 47 unique haplotypes were defined by 61 variable sites. A median-joining (MJ) network (Fig. 3) illustrated the relationship between Eurasian lynx haplotypes and the four haplogroups of South, West 1, West 2, and East. We identified four haplotypes from seven samples in Kazakhstan, including H36 [TIEN3 (Northern Tien Shan Mountains), SAUR1 (Saur Mountains), and AKML1 (Akmola region of northern Kazakhstan)], and three new haplotypes of H45 [TIEN1 (Northern Tien Shan Mountains) and SAUR2 (Tarbagatai Mountains)], H46 [TIEN2 (Northern Tien Shan Mountains)], and H47 [ALTI1 (Altai Mountains)] (Table 2). See the description of localities in Supplementary Table S1.

**Table 2:** Suggested subspecies of Eurasian lynx (*L. lynx*) and its haplotype numbers and relative clades of the control region (CR).

Subspecies	Rueness et al.^8^	Lucena-Perez et al.^9^	Behzadi et al.^10^	This study	
	CR	Whole mitogenome	CR	CR	Cyt *b*
*L. l. isabellinus*	South	Haplogroup 1	South	**H46** (TIEN2)	**HC2** (TIEN2)
*L. l. lynx*	West	Haplogroup 2	West 1	–	
		Haplogroup 3	West 2	–	
*L. l. wrangeli*	Northeast	Haplogroup 4	East	**H36** (TIEN3, SAUR1, AKML1) **H45** (TIEN1, SAUR2) **H47** (ALTI1)	**HC1** (TIEN1, TIEN3, SAUR1, SAUR2, ALTI1, AKML1)
		Haplogroup 5			

**Figure 4:**
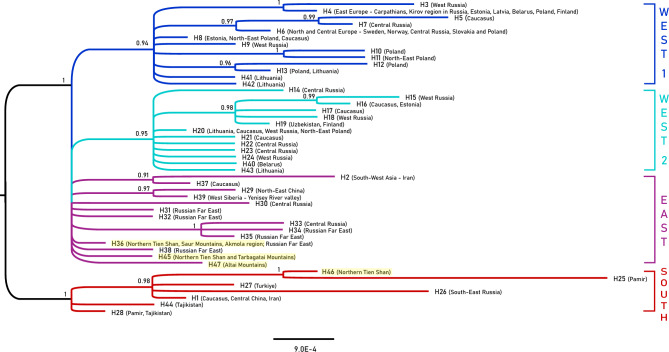
Phylogeny of Eurasian lynx (*Lynx lynx*) based on partial mitochondrial control region sequences. Consensus tree from Bayesian analysis of 47 haplotypes using the HKY substitution model. Samples from Kazakhstan are highlighted in yellow. Numbers at branches indicate support from posterior probability. The colours of the lines correspond to clades: blue = West 1, turquoise = West 2, purple = East, red = South.

The phylogenetic analysis (Fig. 4 and Supplementary Fig. S2) showed the same groups as Behzadi et al.^10^, including a southern group (posterior probability (PP) = 1 and ultrafast bootstrap support (BS) = 93) (South clade) and a northern group comprising samples from Northern Eurasia with one paraphyletic Asian group (East clade) and two monophyletic European groups (West 1 and West 2). The new CR sequences from Kazakhstan belonged to both the southern and the northern groups. H36, H45, and H47 belonged to the East clade, while H46 belongs to the South clade.

In the analysis with the short alignment of cyt *b* (407 bp), only four variable sites (S = 4) existed between trimmed sequences (107 sequences, Figure S7), with two haplotypes (HC2 and HC5) from the southern group (including trimmed cyt *b* sequences of haplogroup 1 and haplogroup 6 based on the complete mitochondrial genome) and three others (HC1, HC3, and HC4) from the northern group (including haplogroups 2–5 of the complete mitochondrial genome). Only two cyt *b* haplotypes were recorded for the Kazakhstani samples, including HC2 from the TIEN2 (Northern Tien Shan Mountains), and HC1 for the other samples (Northern Tien Shan Mountains, Saur-Tarbagatai Mountains, and Akmola region of northern Kazakhstan). HC1 was identical to haplogroups 2–5 of Lucena-Perez et al.^9^ and Bazzicalupo et al.^3^, but HC2 was identical to haplogroup 1. The haplotype from the Balkan region (haplogroup 1 of Lucena-Perez et al.^9^ and Bazzicalupo et al.^3^) was the same as the South clade haplotype (HC2 haplotype). The four other haplogroups of Lucena-Perez et al.^9^ (haplogroups 2–5) were included in the northern group.

## Discussion

We conducted a comprehensive scientific investigation and examined the age, sex, and geographic variability in the lynx skull and mandible morphometric traits within the eastern region of its distribution, specifically with new data from Kazakhstan. The study involved a meticulous phylogeographic analysis of both the morphological characteristics and sequences of mtDNA. These analyses provide valuable insights into the geographic variability and phylogenetic relationships of Eurasian lynx populations and their current systematic status. The results obtained may help in creating lynx conservation strategies considering their and other mammals’ distribution boundaries, migration routes and other features of ecology^26,29^ and biology^30^.

### Variability in morphometric parameters

Our results employing PCA enabled us to suppose that the geographical variations in the cranium of the examined lynx specimens were more prominent than the variations observed in the mandible. This discrepancy in variability between the cranium and mandible has also been observed in other mammalian species^31^. To understand such morphological variability, conducting further research involving larger sample sizes is needed.

### Sexual variability

Sexual variation in lynx populations manifests as size disparities, with males exhibiting greater dimensions than females^32,33^. Skull sexual dimorphism of the lynx can be corrected by Burnaby projection based on simple allometry. Size correction enables us to suggest that differences between males and females in the studied populations are consistent in the size and are related to size difference allometry.

Among carnivorous mammals, disparities in physical traits such as skull size, canine and molar dimensions are observed, with felids displaying the most prominent sexual dimorphism^34^. Pronounced sexual variation in large felid species is particularly evident in those with a polygamous social structure and is believed to be associated with their reproductive behaviour, specifically competition between males for access to females^35,36^. Conversely, species that adopt a solitary lifestyle demonstrate less prominent sexual variation, as observed with the snow leopard (*Panthera uncia*)^34^ and the Eurasian lynx^26^.

### Genetic variability

It is possible that there are three subspecies of lynx (Turkestan lynx *L. l. isabellinus*, Altai lynx *L. l. wardi*, and northern lynx *L. l. lynx*) in Kazakhstan^5^. Based on Behzadi et al.^10^, they include the southern subspecies (South clade, *L. l. isabellinus*), eastern *L. l. wrangeli* (East clade) and western *L. l. lynx* (West 1 and West 2 clades). Two of these subspecies are distinguished in the studied regions in the south, east and northeast of Kazakhstan as *L. l. isabellinus* (H46) and *L. l. wrangeli* (H36, H45, and H47). Previous morphometric studies have suggested the existence of the Turkestan lynx (*Lynx lynx isabellinus*, which is smaller in size and rare) and the Altai lynx (*Lynx lynx wardi*, larger in size and more common) in the Tien Shan Mountain and Altai Mountain regions^5,22^. Some researchers speculate that the Irtysh River potentially acts as a barrier between these regions^7^, while others suggest that the Alakol Basin in the south to the Irtysh River and north to the Zhetisu Alatau and Tien Shan is the barrier between the ‘southern’ and ‘northern’ (northeastern) lynx populations^28^.

Our samples cover both the Altai Mountain region (possible habitat of the Altai lynx) and the Tien Shan Mountain region (one of the possible habitats of the Turkestan lynx)^27^, as well as habitats between the Irtysh River and Alakol Lake Valley—the Saur and Tarbagatai Mountains. It appears that the recovered CR haplotypes of the Altai lynx are identical to the *L. l. wrangeli* (H36, H45, and H47) found in northern Kazakhstan, Altai Mountain, Saur and Tarbagatai Mountains, and Tien Shan Mountain, while the Turkestan lynx (*L. l. isabellinus*) haplotypes are observed only in the Tien Shan Mountain (H46) and may occur up to the Zhetisu Alatau south of the Alakol Lake Valley. Therefore, both subspecies, *L. l. wrangeli* and *L. l. isabellinus*, exist in Tien Shan Mountain. Furthermore, based on the whole-genome sequencing data of Lucena-Perez et al.^9^ and Bazzicalupo et al.^3^, two haplogroups [HG4 (‘Eastern 2’ from Primorsky Krai and Yakutia) and HG5 (‘Eastern 1’ from Mongolia, Tuva, Primorsky Krai, and Yakutia)] were identified for the distribution area of Siberian lynx and Altai lynx. These data also suggested that these two haplogroups, ‘Eastern 1’ and ‘Eastern 2’, are closely related and are the last two separated haplogroups of the Eurasian lynx, and there is no strong evidence of a geographical border between them, as both haplogroups were identified in Primorsky Krai and Yakutia. If further genomic studies confirm that the Siberian lynx (*L. lynx wrangeli* Ognev, 1928) and Altai lynx (*Lynx lynx wardi* Lydekker, 1904) are identical and share the same haplotypes, then *L. lynx wardi* takes priority over *L. lynx wrangeli*, meaning it may be possible to propose using *L. lynx wardi* for the whole eastern subspecies, i.e., the East clade based on the CR dataset as well as both ‘Eastern 1’ (HG5) and ‘Eastern 2’ (HG 4) haplogroups based on the whole-genome sequencing data.

The southern subspecies (*L. l. isabellinus*) has not significantly expanded its range compared to its historic distribution, occurring in the geographic area where it originated in southern Eurasia^10^. This is supported by the mismatch distribution analysis, which suggested an expansion northwards after the LGM, mostly between 8 and 4 thousand years ago, following the separation of the southern group (South clade *L. l. isabellinus*) and the northern group (West 1, West 2, and East clades)^10^. Additionally, it seems that the East clade has extended its range to Iran (a common habitat of *L. l. isabellinus*) with the distribution of its CR haplotype H2 and to eastern Europe (a common habitat of *L. l. lynx*) with haplotype H39.

**Figure 5:**
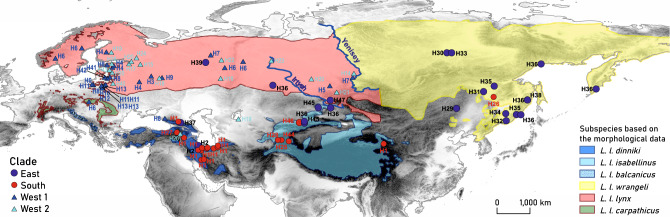
Distribution of subspecies of *L. lynx* based on the morphological data and CR sequences. Yellow *(L. l. wrangeli*), pink (*L. l. lynx*), green (*L. l. carpathicus*), light blue (*L. l. isabellinus*), blue (*L. l. dinniki*), and dotted light blue (*L. l. balcanicus*) polygons correspond to the distribution based on the six subspecies suggested by the IUCN Cat Specialist Group^7^, which are modified from the distribution map of *L. lynx*^10,25^. Circles and triangles represent samples sequenced for the relative clades of the control region (CR). Different shades of grey represent different altitudes. The background hillshade was made using the Shuttle Radar Topography Mission (SRTM) elevation model (http://srtm.csi.cgiar.org) in QGIS v.3.10; country boundaries were downloaded from the DIVA-GIS dataset (http://www.diva-gis.org/Data), and the layout was made in QGIS v.3.10.

Gugolz et al.^16^ proposed a postglacial recolonization scenario for the northern lynx in Europe. Rueness et al.^8^ highlighted that the most ancient lineage of lynxes can be traced back to Central Asia, suggesting that the origin of Eurasian lynx populations can be attributed to Central Asia, followed by a subsequent expansion towards northwestern Siberia and Scandinavia from the Caucasus and towards north-eastern Siberia from an eastern refugium after the LGM (within 26.5–19 thousand years ago). The Tien Shan, Altai, Pamirs, and Himalayas likely served as the contact zone for the South clade populations in the southern areas with other clades (West 1, West 2, and East) due to postglacial range expansions (Fig. 5).

Phylogenetic trees generated using the UPGMA method based on morphometric data (Fig. 2) and mtDNA sequences (Figs. 4 and 5), calculated through pairwise genetic distance analysis between individuals, have unveiled that Altai seems to be the edge of distributional range of the different clades that meet there. Migration corridors may have been in the Saur-Tarbagatai region and along the Tien Shan ranges.

Based on the MJ network (Fig. 3) and phylogenetic tree (Fig. 4), the southern group (*L. l. isabellinus*) is separated from the northern group, which includes the East clade to the east and West 1 and West 2 to the western side of the Yenisey River. There are some barriers between them, such as mountains in the south between the southern group and the northern group, and the Yenisey River between the East and Western clades (West 1 and West 2). All these clades are present in Kazakhstan, where the representatives of the three clades reach each other in the contact zone. In East Kazakhstan, the Saur-Tarbagatai region and the Tien Shan ranges may act as a corridor to exchange haplotypes such as H26 of the South clade in eastern Asia in the main geographic distribution of the East clade, as well as the existence of haplotypes of the East clade on the western side of the Yenisey River such as H36 and H39 in the geographic distribution areas for *L. l. lynx* (West 1 and West 2).

Overall, phylogenetic analysis identified the Altai samples as distinct haplotypes, coinciding with the Siberian lynx. As haplotypes of the West 1 clade (H5 and H7) and West 2 (H14, H20, and H21) from the western area of Altai Republic and Krasnoyarsk Krai were previously identified in the Western clades (Supplementary Table S4), the distribution border of the northern lynx also reaches these areas (Fig. 5). The proximity to northeastern populations by the Altai population may be attributed to the range expansion. In contrast, the Turkestan lynx, based on sample TIEN2 from the Northern Tien Shan, is recognized as an isolated subspecies. Consequently, the Tien Shan and Altai regions may be considered a contact zone for subspecies, particularly the southern (*L. l. isabellinus*) and eastern (proposed here as *L. l. wardi*) subspecies. The border between the southern subspecies (*L. l. isabellinus*) and the northern ones, namely, the northern (*L. l. lynx*) and Altai (*L. l. wardi*) lynxes, lies within this contact zone.

### Geographic variability

The expression of clinal geographic morphological variation within the examined region is relatively weak. Our investigation did not reveal a notable size gradient in lynxes from north to south, which is commonly observed in other mammalian species, such as foxes^37^ and wolves^38^.

Furthermore, the structuring of skull shape variability was poor. Nevertheless, we identified some distinctions in cranial morphology between the Altai and Tien Shan populations. Specifically, the samples exhibited significant differences in the breadth of the zygomatic bone, one of the key indicators of subspecies differentiation in mammals^39–41^. Positioned geographically between the Altai and Tien Shan regions, the Saur-Tarbagatai population also displayed intermediate cranial characteristics, aligning with its transitional location. As expected, there was minimal discernible variation between the Altai and Tien Shan populations in terms of mandible features.

The interesting findings of our study were the distinctions in qualitative characteristics between the Altai and Tien Shan populations. Specifically, within the examined samples of Altai lynxes, we observed a metaconid (morphotype 3), which is a tubercle on the lower teeth row molar (M_1_), in 55.6% of cases. Intriguingly, the metaconid was absent in 66.7% of samples from the Northern Tien Shan, aligning closely with the findings of Kitchener et al.^7^, who reported the absence of metaconid in 75% of Turkestan lynxes. This suggested that the presence or absence of metaconids in lynxes is a variable trait with inconsistent occurrence^42,43^. Over the Middle Pleistocene period, lynxes exhibited a gradual decline in this structure^44^, and Bonifay^45^ and Testu^46^ hypothesized that this gradual reduction may be linked to the development of sharper dentition among lynxes. In our samples, we observed a gradual decline in metaconid presence from the north to the south (Altai—Tien Shan—Pamir). In particular, 50.0% of our Tien Shan lynx samples have an inflection of the paraconid enamel (morphotype 2, which is one step away from the metaconid structure). *Lynx lynx* is the only species in the *Lynx* genus that occasionally has a second lower molar (M_2_) (from 3 up to 27% of individuals depending on the region), while the metaconid within the species is well developed^47^. As other lynx species have less developed M_1_ structures (morphotypes 1 or 2), it is hypothesized that the selection pressure during the Pleistocene acted directly on the phenotype, i.e., the decline of this dentition trait among northern lynxes and the widespread appearance of metaconids in *L. l. lynx* populations could be the result of selection pressure and reversal of dental structure^47,48^. More research is needed to understand the role of dentition traits in lynx phylogeny.

The presence of skull shape variations and a distinct qualitative trait suggests that the studied populations display partial isolation from one another. However, the results obtained from comparative morphometric analysis do not support the classification of Altai lynx populations as a separate subspecies but indicate that the cranial and mandibular characteristics of Altai lynx overlap with those of the Turkestan lynx specimens (Fig. 1). Nonetheless, it is important to highlight the presence of a unique set of morphological characteristics specific to each population, which may reflect ecological peculiarities associated with their respective habitats^9^, which requires further studies including larger sampling.

Consequently, the populations inhabiting the Tien Shan and Altai regions exhibit distinctive morphological traits. The Altai lynx population exhibits morphological similarities with samples from the northeastern region of the Eurasian lynx population, specifically the Krasnoyarsk Krai and Novosibirsk Region, while also displaying some overlap with samples from the Northern Tien Shan.

To preserve the remarkable morphological and ecological diversity observed, it is crucial to establish protective measures for the lynx populations in the eastern distribution range, especially in the Tien Shan Mountain region, where the lynx is vulnerable, as well as Altai lynx populations and the transitional Saur-Tarbagatai Mountain region. A continued comprehensive and in-depth examination of the morphological characteristics of the animals occupying the study area is warranted to further enhance our understanding.

## Conclusion

Our comprehensive study of lynx populations in Central Asia, including new data from Kazakhstan, has provided valuable insights into their geographic variability, genetic diversity, and phylogeographic structure. Through morphological analysis, we determined that there are minimal geographical variations in the cranium from north to south Kazakhstan, but some distinctions in cranial morphology are present in the Turkestan and Altai lynx populations.

Skull size and dimension gradient were more prominent than those observed in the mandible. Sexual variation was also observed, with males exhibiting greater dimensions than females, although the relative proportions of the cranium remained consistent between the sexes. Additionally, qualitative traits such as the presence or absence of specific dental structures differed between these populations. Further studies involving larger sample sizes are necessary to understand these qualitative and quantitative morphological characteristics.

In terms of genetic variability, distinct haplotypes were identified, with the Northern Tien Shan population (CR haplotype H46) showing haplotypes of the southern Turkestan lynx. Three other CR haplotypes (H47, H45, and H36) correspond to the East clade and suggest minor genetic variations within the lynx populations in Kazakhstan and eastern Asia. The interconnectivity between populations in Central Asia, as evidenced by the presence of two different clades in Kazakhstani lynx populations, highlights the continuity and genetic exchange within the region, including the Tien Shan mountainous area.

Our findings of Kazakhstani haplotypes support the classification of Altai lynx (*L. l. wardi*) as a separate subspecies from Turkestan lynx (*L. l. isabellinus*). Genetic analyses based on mtDNA sequences have revealed shared haplotypes between Altai and Siberian lynxes, suggesting that these two belong to the same East clade. Geographic territories, such as mountains (Altai and Saur-Tarbagatai Mountains), rivers (Yenisey and Irtysh Rivers) and lakes (Alakol Lake Valley), influence population dispersion and may serve as partial barriers to dispersal. To gain a comprehensive understanding of the phylogeography and systematics of lynxes in Central Asia and Eurasia as a whole, further investigations incorporating the complete genome and larger morphological sample sizes of these populations are needed.

## Materials and methods

### Data acquisition

**Figure 6:**
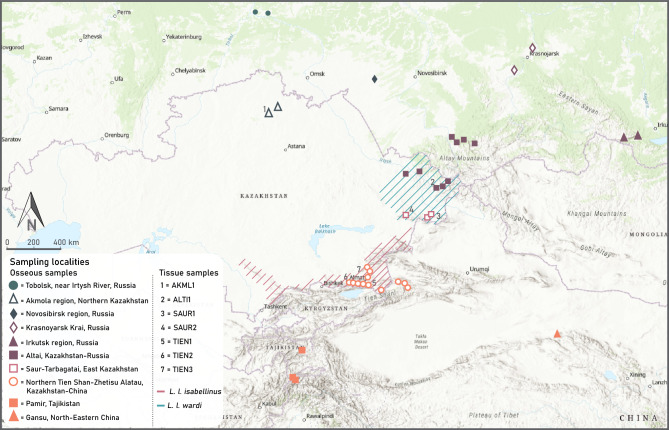
Sample collection regions for morphometric analysis (osseous samples) and phylogenetic analysis (tissue samples) in Central Asia and distribution limits in Kazakhstan. Additionally, an osseous sample from Estonia was used for morphometric analysis as a representative of a typical northern lynx specimen. The distribution border between *L. l. isabellinus* and *L. l. wardi* is located around the Alakol Lake Valley and the Irtysh River. The background hillshade was made using the Shuttle Radar Topography Mission (SRTM) elevation model (http://srtm.csi.cgiar.org) in QGIS v.3.10; country boundaries were downloaded from the DIVA-GIS dataset (http://www.diva-gis.org/Data), and the layout was made in QGIS v.3.10.

Our materials consisted of 40 cranium and 42 mandibular samples from 10 and 11 localities, respectively, for morphometric analyses (Fig. 6). For phylogenetic analysis, we collected seven tissue samples from dead animals of different Eurasian lynx populations in Kazakhstan between 2016 and 2021 (Fig. 6). These samples were legally collected from dead animals during expeditions and from the collection fund of the Institute of Zoology (Almaty, Kazakhstan). For details on the tissue sample localities and types, see Supplementary Table S1.

For morphometric analyses, we studied osseous samples from various zoological collections, including the Institute of Zoology (Kazakhstan), the Biological Museum of al-Farabi Kazakh National University (Kazakhstan), the Research Zoological Museum at Lomonosov Moscow State University (Russia), and the Zoological Institute of the Russian Academy of Science (Russia). We also included samples from private collections of taxidermists. Descriptions of the skulls analysed are provided in Supplementary Table S2.

### Cranial measurements

We measured 27 cranial characteristics (Supplementary Fig. S3) and 21 mandibular characteristics (Supplementary Fig. S4), including 17 dental measurements from the upper and lower teeth rows, following standard methodology^49–51^. The measurements were recorded using a Vernier calliper with a precision of 0.1 mm. The descriptions and acronyms for these measurements are listed in Supplementary Table S3.

Initially, we grouped the specimens by age (juvenile, subadult, adult) and sex (male, female) (Supplementary Fig. S5). Juvenile lynx are individuals in the first year of life (0–1 year old); subadults are females in their second (1–2 years old) and males in their second and third years (1–3 years old); adults are all older lynx (over 2 years old females and over 3 years old males)^52^.

To identify the age and sex of specimens that were not labelled in the collection records, we relied on the criteria outlined by Garcia-Perea et al.^53^ and Klevezal^54^. Juveniles (due to underdeveloped skulls) and specimens with cranium or mandible fractures were excluded from statistical data processing.

From all specimens considered, the age of 29 was indicated in the collection records. For the remaining specimens, we estimated their approximate age based on the closure of the coronal suture and the development of the sagittal and occipital crests. The juveniles were identified by the open apical foramen in the canine root^55^, the presence of forming third premolar (P_3_) and molar (M_1_) in the lower teeth row, and underdeveloped sagittal and occipital crests. The coronal suture was open in juveniles, partially closed in subadults, and fully closed in older adults. The sagittal crest in males of the subadult group was also comparably not developed, which was a characteristic of female adult specimens used for analysis^50,56^. Sexual dimorphism in the cranial structure became apparent through the progressive formation of ridges over time (see Supplementary Fig. S5 for more details). These age-related alterations aligned with patterns observed in other species within the same genus, where there was a relative augmentation in the facial region of the skull alongside the emergence of postorbital compression^5^. In adult males, sagittal and occipital crests develop^50^, and the coronal suture is closed in both males and females. Based on these age criteria, we assigned 9 specimens to the juvenile group, 13 to the subadult group (up to 2 years for females and 3 years for males^5^), and 32 to the adult group.

Similarly, 28 adult and subadult specimens were of known sex (16 males and 12 females), while the rest were analysed based on a comparison of sagittal and occipital crest characteristics^54^, total skull length, and zygomatic width between the two sexes^53^. Thus, excluding juveniles, we analysed the other specimens and assumed 6 of them to be males and 11 to be females; in total, there were 22 males and 23 females.

The analysis of cranial and mandibular measurements was conducted separately to optimize the ratio of the number of measurements to the number of specimens and to maximize the number of specimens included in the analysis. All analyses were based on log-transformed data to linearize allometric relationships between linear measurements^57–59^.

### Statistical analysis of morphological variables

To correct the effect of age and sex variation, Burnaby’s method^60^ was employed. This procedure eliminates the effects of growth allometry from multivariate data by projecting data points onto a subspace that is orthogonal to the age and sex vectors^58,59,61–66^. The new subspace has two dimensions less than the original space. The age and sex vectors were derived as the first eigenvector of the covariance matrix for the age and sex effect^59,67–69^. In other words, the Burnaby projection has allowed for the elimination of sexual dimorphism and age differences by calculating the data as vectors that connect centroids between female and male samples, as well as adult and subadult samples.

We estimated the effects of age, sex, and geographical variation with multivariate analysis of variance (general linear model, GLM) on both log-transformed and Burnaby-transformed data using age (AGE), sex (SEX) and locality (LOC) as categorical predictors.

We applied principal component analysis (PCA) and the unweighted pair group method with arithmetic mean (UPGMA) cluster analysis with Burnaby-transformed data to examine the pattern of variation between individual specimens and between groups of individuals from the same sampling localities, respectively. UPGMA was performed using the matrix of Mahalanobis quadratic distances between group centroids. We included samples with at least 2 specimens in this analysis. A total of 7 localities were included in the cluster analysis for both cranium and mandible samples. For cranium data, the following localities were used: Tobolsk, Russia; Akmola region, Kazakhstan; Krasnoyarsk Krai, Russia; Altai Kazakhstan and Russia; Northern Tien Shan, Kazakhstan and China; Irkutsk region, Russia; Saur-Tarbagatai, Kazakhstan. For mandible data, the following localities were used: Tobolsk, Russia; Akmola region, Kazakhstan; Krasnoyarsk Krai, Russia; Altai Kazakhstan and Russia; Northern Tien Shan, Kazakhstan and China; Irkutsk region, Russia; and Pamir, Tajikistan (Fig. 6).

Two localities of interest (Altai (sample number 7) and Northern Tien Shan (sample number 9)) were compared with individual sex- and age-adjusted traits using a Bonferroni-adjusted t test for pairwise comparisons. Cluster means were compared for individual characteristics using the Mann–Whitney U test with Bonferroni correction^70^.

To test for size variation in cranium and mandibular dimensions based on latitude, we analysed the correlation between the condylobasal length of the cranium and the latitude where the specimen was first found, as well as the correlation between the mandible length and the latitude. The condylobasal length and mandible length approximate body size variation well^71–73^. For the analysis, we used logarithmic data and included only adult specimens, and the analysis was performed separately for males and females to avoid a sexual dimorphism impact on the results.

We analysed the dental features of mandibles, specifically comparing three morphotypes of lower first molar M_1_^53^. Type 1 M_1_ did not have a mesiolingual cusp (metaconid), type 2 had an inflection in the paraconid enamel, and type 3 had the metaconid present on the cingulum (as shown in Supplementary Fig. S6). We estimated the frequency of occurrence in samples 7 and 9, Altai and Northern Tien Shan.

### DNA extraction, amplification and sequencing

We obtained seven tissue samples (hair epithelium, muscle and tooth tissues), preserved them in 96% ethanol and stored them at − 20 °C before DNA extraction. Supplementary Table S1 lists the tissue type and localities relevant to osseous sample types and localities, while Supplementary Table S4 lists all the ID, source and reference numbers of tissue samples obtained in this study and downloaded from NCBI GenBank and contains information about year and geographical origin, as well as the possible clades of the samples analysed.

Whole genomic DNA was extracted from tissue samples using the DNeasy Blood & Tissue Kit (QIAGEN) following the manufacturer’s instructions with a slightly modified protocol^8^. Polymerase chain reaction was performed to amplify a 613 base pair (bp) fragment of the control region (CR) using mtU (5´-CTTTGGTCTTGTAAACCAAAAAA-3´) and R3 (5´-TAAGAACCAGATGCCAGGTA-3´) primers, as well as 376 bp of cyt *b* using Cytb-1 (CCAATGATATGAAAAACCATCGTT) and Cytb-2 (GCCCCTCAGAATGATATTTGTCCTC) primers^74^. Amplifications were performed in 50 μl volumes containing 2 μl of genomic DNA, 2 μl of forward primer, 2 μl of reverse primer, 25 μl of NEXpro™ qPCR Master Mix (SYBR), and 19 μl of ddH_2_O.

Thermocycling was performed in a *Biometra TAdvanced PCR* (Analytik Jena) thermocycler using initial denaturing at 95 °C for 4 min followed by 40 cycles of 30 s at 94 °C, 35 s at 55 °C, and 50 s at 72 °C and a final extension at 72 °C for 5 min. Sanger sequencing was performed using the BigDye Terminator Cycle Sequencing kit v.3.1 (Applied BioSystems), and electrophoresis of the purified sequencing product was carried out on a Maxicell Primo EC340 on a gel electrophoresis system.

### Phylogenetic analysis

The mtDNA sequences were edited with SeqScape v.2.6 (Applied Biosystems) and aligned with 85 previously published mtDNA sequences from GenBank using the Clustal W algorithm^75^, implemented in Mega v.5^76^, checked visually and trimmed to match the sequence length of our sequences.

DnaSP^77^ was used to find the number of haplotypes, and the distribution map of haplotypes was made in QGIS v.3.10. An MJ network was constructed using PopART v.1.7^78^ with the default settings (Fig. 3 for CR and Supplementary Fig. S7 for cyt *b*). For the phylogenetic analysis, we included only one representative of each haplotype. The Hasegawa-Kishino-Yano substitution model (HKY)^79^ was selected as the best model of nucleotide substitution based on the Bayesian Information Criterion (BIC) scores using jModelTest v.0.1.1^80^. A Bayesian phylogenetic tree was constructed in MrBayes v.3.2.7a^81^ using two independent runs of four Markov chain Monte Carlo (MCMC) runs that were run simultaneously for 10,000,000 generations and sampling every 1,000 generations. The first 25% of the sampled trees and estimated parameters were discarded as burn-in. Convergence of the model parameters was monitored using the program Tracer v.1.7.1^82^. The consensus phylogenetic tree was then edited in FigTree v.1.4.4 (http://tree.bio.ed.ac.uk/software/figtree/). For the maximum-likelihood tree reconstruction, we obtained branch supports with ultrafast bootstrapping^83^ with 2000 replicates implemented in IQ-TREE software v.2.2.0^84^, and the consensus phylogenetic tree was edited in FigTree v.1.4.4 (http://tree.bio.ed.ac.uk/software/figtree/).

### Supplementary Information

Supplementary Information.

Supplementary Figure S1.

Supplementary Figure S2.

Supplementary Figure S3.

Supplementary Figure S4.

Supplementary Figure S5.

Supplementary Figure S6.

Supplementary Figure S7.

## Data Availability

The control region and cytochrome *b* dataset generated and/or analysed during the current study are available in the GenBank repository (https://www.ncbi.nlm.nih.gov/genbank/) under accession numbers: MK229198–MK229293. All morphological data generated or analysed during this study are included in this published article and its Supplementary Information files.
