# Supplementary material for: Insights into subspecies classification and conservation priorities of Central Asian lynx populations revealed by morphometric and genetic analyses
Source: Sci Rep. 2024 Mar 2;14:5186. doi: 10.1038/s41598-024-55807-x (PMC10908838; doi:10.1038/s41598-024-55807-x)
Supplement: Supplementary file 1 — Supplementary Information. [file 41598_2024_55807_MOESM1_ESM.pdf]

Morphometric and genetic analyses of Central Asian lynx populations: insights into subspecies classification and conservation priorities

Supplementary Figure legends

Supplementary Figure S1. Distribution of variance (%) among the axes of the principal components: A) skull, B) mandible.

Supplementary Figure S2. Phylogeny of Eurasian lynx (*Lynx lynx*) based on partial mitochondrial control region sequences. Consensus tree from the maximum-likelihood tree reconstruction of 47 haplotypes using the HKY+F substitution model. Numbers at branches indicate support from ultrafast bootstrap. The colors of the lines correspond to clades: blue = West 1, turquoise = West 2, purple = East, red = South.

Supplementary Figure S3. 19 skull and 8 dental (upper teeth row) characteristics used for analysis (see Table S3 for descriptions).

Supplementary Figure S4. 12 mandibular and 9 dental (lower teeth row) characteristics used for analysis (see Table S3 for descriptions).

Supplementary Figure S5. Comparison of cranium in age and sex groups among previously studied individuals[85]: A) Lateral view. i) An adult male (No. S-134442, RUS), with 1 – prominent external sagittal crest; ii) An adult female (No. 879, KAZ), with 2 – partial sagittal crest or its absence; 3 – iii) a juvenile (No. 56, KAZ), with absence of external sagittal crest. $\bar{X}$ = mean, total skull length among individuals of different sex and age. B) Dorsal view. i) An adult male cranium (No. S-134442, RUS); ii) A subadult male (No. 659, KAZ), with a developing sagittal crest; iii) a juvenile (No. 56, KAZ). 1 – Coronal suture; 2 – Sagittal suture.

Supplementary Figure S6. M_1 morphotypes. Type 1 – M_1 with no metaconid; Type 2 – M_1 with an inflection in the paraconid enamel; Type 3 – M_1 with the metaconid.

Supplementary Figure S7. Median-joining network based on the trimmed Cyt *b* gene depicting the relationships between the main six haplogroups described by Bazzicalupo et al.[3] and Lucena-Perez et al.[9] for Eurasian lynx. Black small dots represent missing haplotype; circle sizes are proportional to haplotype frequencies; numbers are haplotype numbers. Kazakhstani haplotypes (HC1 and HC2) highlighted in yellow color.

Supplementary Tables

Supplementary Table S1. Specimens' localities (skulls and mandibles for morphometric analysis, tissue samples for phylogenetic analysis).

No	Specimen location	Skulls	Mandibles	Tissue samples
1	Estonia, Northern Europe	1 sample, male	1 sample, male	–
2	Tobolsk, near Irtysh, Russia	2 samples, sex unknown	2 samples, sex unknown	–
3	Akmola region, North Kazakhstan	2 samples, females	2 samples, females	AKML1, muscle
4	Novosibirsk region, Russia	1 sample, female	1 sample, female	–
5	Krasnoyarskiy krai, Russia	2 samples, 1 – male, 1 – unknown	2 samples, 1 – male, 1 – unknown	–
6	Irkutsk region, Russia	2 samples, 1 – male, 1 – female	2 samples, 1 – male, 1 – female	–
7	Altai (Gorniy Altai, Russia, South Altai, East Kazakhstan)	9 samples, 1 – male, 2 – female; 6 – unknown	9 samples, 1 – male, 2 – female; 6 – unknown	ALTI1, muscle
8	Saur-Tarbagatai, East Kazakhstan	2 samples, sex unknown	1 sample, sex unknown	2 samples (SAUR1 – skin epithelium, SAUR2 – muscle)
9	Northern Tien Shan and Zhetisu Alatau, Kazakhstan and China	18 samples; 8 – males; 4 – females; 6 – unknown	18 samples, 8 – males, 5 – females; 5 – unknown	TIEN1 (hair epithelium), TIEN2 (muscle), TIEN3 (tooth)
10	Pamir, Tajikistan	1 sample, male	3 samples, 2 – male, 1 – female	–
11	Gansu, Tibet	–	1 sample, male	–

Supplementary Table S2. Information on specimens measured including skull (S) and mandible (M).

ID	Sex	Age	Date	Place of collecting/finding	Collector	S	M
**Collection of Institute of Zoology, Almaty, Kazakhstan**							
7/11809	Male*	Uncertain. Possibly subadult*	February, 1957	Estonia	Estonian AS USR	+	+
8/13075	Male	Uncertain. Possibly adult	February 1934	Tunkin Valley, near Irkutsk city, Russia	A. Sludskiy	+	+
5/11136	Female	Uncertain. Possibly adult	February 1934	Tunka, near Irkutsk city, Russia	A. Sludskiy	+	+
14544	Uncertain. Possibly	Uncertain. Possibly subadult	1959	Ile Alatau Mountains, South-East Kazakhstan	A. Sludskiy	+	+

	male						
6/11137	Uncertain. Possibly female	Uncertain. Possibly subadult	1924	Katon Karagay district, East Kazakhstan	?	+	+
16/18959	Male	Uncertain. Possibly subadult	November, 2021	Saur Mountains, Temirsu Gorge, East Kazakhstan	N. Bizhanova	+	+
13/18956	Uncertain. Possibly female	Uncertain. Possibly adult	June 2014	Kungey Alatau, Kolsai Kolderi NP, SE Kazakhstan	A. Grachev	+	-
14/18957	Uncertain. Possibly female	Adult	May 2019	Upper streams of Shelek River, SE Kazakhstan	A. Grachev	+	+
15/18958	Uncertain. Possibly female	Uncertain. Possibly subadult	November, 2021	Katon Karagay district, East Kazakhstan	N. Bizhanova	+	+
**Collection of Section of Theriology at Zoological Museum of Lomonosov State University, Moscow, Russia**							
S-94178	Uncertain. Possibly female	Uncertain. Possibly adult	January 1965	Almaty region, Bartogay gorge, SE Kazakhstan	Gvozdev	+	+
S-46158	Uncertain. Possibly male	Uncertain. Possibly subadult	January, 1948	Gorniy Altai, Russia	Berger	+	+
S-46159	Uncertain. Possibly male	Uncertain. Possibly adult	January, 1948	Gorniy Altai, Russia	Berger	+	+
S-46160	Uncertain. Possibly female	Uncertain. Possibly subadult	January, 1948	Gorniy Altai, Russia	Berger	+	+
S-134442	Male	Adult	August, 1977	Krasnoyarskiy Krai, Krasnoyarskiye Stolby, Russia	A. Zyryanov	+	+
**Collection of taxidermist Safronov Servey, Astana, Kazakhstan**							
1/143	Female	Uncertain. Possibly adult	November 2020	Zerendi District, Akmola region, North Kazakhstan	S. Safronov	+	+
2/037	Uncertain. Possibly male	Uncertain. Possibly adult	November, 2021	Tarbagatai, East Kazakhstan	S. Safronov	+	+
**Biological museum of al-Farabi Kazakh National University, Almaty, Kazakhstan**							
42	Female	Adult	1975	Ile Alatau Mountains, SE Kazakhstan	Zhuyko	+	+
55	Uncertain	Juvenile	1977	Ile Alatau Mountains, SE Kazakhstan	G. Arend	+	+
56	Uncertain	Juvenile	1977	Ile Alatau Mountains, SE Kazakhstan	G. Arend	+	+
483	Male	Adult	February, 1992	Almaty Zoo, SE Kazakhstan	B. Zhuyko	+	+
497	Female	Adult	October, 1992	Bolshiye Boguty, SE Kazakhstan	B. Zhuyko	-	+
525	Male	Adult	September, 1994	Zhetisu Alatau, SE Kazakhstan	Kikimov	+	+
628	Male	Subadult	1999	Ile Alatau Mountains, SE Kazakhstan	B. Zhuyko	+	+
629	Male	Adult	1999	Kungey Alatau, SE Kazakhstan	B. Zhuyko	+	+
650	Male	Adult	December, 2001	Zhetisu Alatau, SE Kazakhstan	B. Zhuyko	+	+
659	Male	Subadult	September,	Ile Alatau Mountains, SE Kazakhstan	B. Zhuyko	+	+

			2002				
695	Female	Subadult	January, 2004	Kokshetau, North Kazakhstan	B. Zhuyko	+	+
718	Male	Adult	September, 2007	Narynkol, Bolshie Kokpan, SE Kazakhstan	B. Zhuyko	+	+
798	Female	Adult	December, 2009	Ile Alatau Mountains, Ushqonyr Gorge, SE Kazakhstan	B. Zhuyko	+	+
831	Female	Adult	January, 2015	Almaty Zoo, SE Kazakhstan	B. Zhuyko	+	+
879	Female	Adult	November 2016	Big Almaty Gorge, Japanese Road, SE Kazakhstan	B. Zhuyko (via Kantarbayev & Bespalov)	+	+
**Zoological Institute of Russian Academy of Science, Saint-Petersburg, Russia**							
9415 (№330.1916)	Uncertain	Uncertain. Possibly juvenile	November 1914	Saur Mountains, Zaisan, Semey area, East Kazakhstan	V. E. Martino	+	+
27518 (№928)	Uncertain. Possibly female	Uncertain. Possibly adult	1961	Saur Mountains, northern slopes, Zaisan, East Kazakhstan	Zoological Institute RAS	+	-
32132	Male	Juvenile	December 1983	10 km from Oskemen, Altai, East Kazakhstan	Kruglov &Vereschagin	+	+
32133	Female	Adult	December 1983	10 km from Oskemen, Altai, East Kazakhstan	Kruglov &Vereschagin	+	+
32177	Male	Adult	October 1981	Monastyri raven, East Kazakhstan	Kruglov &Vereschagin	+	+
1289	Uncertain. Possibly female	Uncertain. Possibly subadult	1877	Tobolsk Governorate, Irtysh River Basin, Russia	Poljakow	+	+
1359	Uncertain. Possibly female	Uncertain. Possibly adult	1877	Tobolsk Governorate, Irtysh River Basin, Russia	Poljakow	+	+
20478 (№9-1930(11))	Uncertain. Possibly female	Uncertain. Possibly subadult	January, 1929	Katon-Karagai district, Arshaty village, along the Krutaya River, left tributary of Bukhtarma River, East Kazakhstan	V. Dauenko	+	+
21853 (№121-1939; №9)	Uncertain. Possibly female	Uncertain. Possibly adult	November 1930	Krasnoyarsk krai, Balakhtinsky District, Western Sibir, Daurskoye village, along the left riverside of Yenisey River, Russia	E. Yakovlev	+	+
27129	Female	Uncertain. Possibly adult	March, 1959	Novosibirsk region, Kargat village, Russia	Grigoryev	+	+
32152 (№4701)	Female	Uncertain. Possibly subadult	October, 1969	Altai Region, Turochakskiy district, Priteletskaya taiga, Pypsi River, Russia	N.N. Vorontsov	+	+
32153 (№5473-1970-26) (№5478-179-28)	Uncertain. Possibly female	Uncertain. Possibly juvenile	April, 1970	Novosibirsk region, Toguchinsky district, Russia	V. I. Telegin	+	+
1164 (№3208; №4976)	Uncertain. Possibly male	Adult	1880	Ili River, Ghulja (Yining), Tien Shan, Xinjiang, Northwest China	Begel	+	+
1284	Male	Adult	1880	Ili River, Ghulja, Tien Shan, Xinjiang,	Begel	+	+

(№10)				Northwest China			
1287 (№3205; №4978)	Uncertain. Possibly male	Adult	1880	Ili River, Ghulja, Tien Shan, Xinjiang, Northwest China	Begel	+	+
1325 (№3207)	Uncertain. Possibly female	Uncertain. Possibly juvenile	1880	Ili River, Ghulja, Tien Shan, Xinjiang, Northwest China	Begel	+	+
7700 (№3-1902; skin 6187)	Male	Juvenile	February 1901	Dza Chu River (upper Mekong basin), Kham, Tibetan Plateau	P. K. Kozlov	+	+
7702 (№3-1902)	Male	Adult	February 1900	Gansu, Tibetan Plateau, Northwest China	P. K. Kozlov	+	+
9408 (№91-1913)	Female	Adult	1913	Western Pamir, Ishkoshim District, Ishkoshim Range, Mulvoj River, Gorno-Badakhshan Region, Tajikistan	Administration of Pamir detachment	+	+
9409 (№91-1913)	Male	Adult	1913	Western Pamir, Ishkoshim Range, Gorno-Badakhshan Region, Tajikistan	Administration of Pamir detachment	+	+
9412 (№120-1913(1))	Male	Adult	1913	Pamir, Tajikistan	Zoological Institute of RAS	+	+
20371 (skin №22533)	Uncertain. Possibly female	Juvenile	October, 1932	Pamir, Vanj River (right tributary of the Panj River), Vanj District, North-Western Gorno-Badakhshan, Tajikistan	M. Rozanov	+	+
9421 (№13154; skin №275-1910(12))	Male	Juvenile	October, 1908	Mudshik He Gorge (Ye'erpu Gang), to the right of the upper stream of Huang He River (Yellow River), Guide County, Hainan Tibet, Qinghai Province, Northwest China	P.K. Kozlov	+	+

* Note: the sex or age of the sample was not indicated in the labels of collection funds and was determined by the author.

Supplementary Table S3. Measurements of skulls, mandibles and teeth.

No	Abbreviations	Description
**Skull measurements**		
1	TSL	Total skull length
2	CBL	Candilobasal length
3	MPL	Median palatal length
4	OAL	Length from the most rostral point of the orbit to Akrokranion
5	UTRL	Upper tooth row length
6	C^1P^3L	Length from the rostral margin of the upper caninus (C^1) alveolus to the caudal margin of the upper third premolar (P^3) alveolus
7	ZW	Zygomatic width
8	POC	Postorbital constriction

9	IOC		Interorbital constriction
10	MB		Mastoid breadth
11	NCC		Nasal cavities constriction
12	RWP^2		Rostral width at the second premolar (P^2)
13	BBC		Breadth of braincase
14	RWC^1		Rostral width: between the lateral margins of the C^1 alveoli
15	MDW		Maxillar dental width: between the lateral margins of P^4 alveoli
16	ZAL		Length of the zygomatic arch
17	P^3P^4L		Length from the rostral margin of P3 alveolus to caudal margin of the P^4 alveolus
18	MSL		Masseteric scar length
19	MSW		Masseteric scar width
20	C^1	a	Upper caninus C^1 width
21		b	C^1 length
22	P^3	a	Upper third premolar P^3 width
23		b	P^3 length
24	P^4	a	Upper fourth premolar P^4 width without protoconus
25		b	P^4 length
26		c	P^4 width with protoconus
27		d	P^4 protoconus length
**Mandibular measurements**			
1	ML		Mandibular length at angular process
2	JL		Jaw length at coronoid process
3	MDL		Mandibular dental length
4	LJH		Lower jaw height
5	TMA		Temporalis muscle moment arm
6	MMA		Masseteric moment arm
7	JHM_1		Jaw height at M1. Height of the body of the mandible at the caudal margin of the lower M_1 alveolus
8	JHP_3		Jaw height at lower P_3
9	P_3M_1L		Length from the rostral margin of P_3 alveolus to the caudal margin of M_1 alveolus
10	CM_1L		Condyle to M_1 length of jaw
11	JWM_1		Jaw width at M_1
12	CCL		Condyle to canine length of jaw
13	C_1	a	Lower caninus C_1 width
14		b	C_1 length
15	P_3	a	Lower third premolar P_3 width
16		b	P_3 length
17	P_4	a	Lower fourth premolar P_4 width
18		b	P_4 length
19	M_1	a	Lower molar M_1 width
20		b	M_1 rostral length
21		c	M_1 caudal length

Supplementary Table S4. List of the Eurasian lynx sequences used in phylogenetic analysis (obtained by the authors of this study and downloaded from NCBI GenBank). Haplotype labeling was done according to Behzadi et al. (2022) and new haplotypes obtained in this study are H45, H46, and H47.

№	ID No.	Source	Country	Location	Coordinates		Reference	Year	H	Reference No.	Clade
					Latitude	Longitude					
1	GHZV1	AFD*	Iran	Ghazvin, Abik, Saein Dareh	36.35805556	49.14833333	Behzadi et al., 2022	2016	H2	OM743776	EAST
2	GHZV2	AFD	Iran	Ghazvin, Abik, Saein Dareh	36.35805556	49.14833333	Behzadi et al., 2022	2016	H2	OM743777	
3	TIEN3	IZK	Kazakh-stan	Northern Tien Shan, between Ile and Kungei Alatau Mountains, upper stream of Shelek River	43.4176166	78.3062216	This study	2016	H36	OR837124 (CR), OR837131 (Cyt *b*)	
4	TIEN1	IZK	Kazakh-stan	Northern Tien Shan, Kungei Alatau Mountains	42.9727712	78.3252857	This study	2016	H45	OR837122 (CR), OR837129 (Cyt *b*)	
5	SAUR 1	IZK	Kazakh-stan	Saur Mountains	47.1212176	85.4944601	This study	2021	H36	OR837125 (CR), OR837132 (Cyt *b*)	
6	SAUR 2	IZK	Kazakh-stan	Tarbagatai Mountains	47.2294118	82.0894796	This study	2021	H45	OR837126 (CR), OR837133 (Cyt *b*)	
7	ALTI1	IZK	Kazakh-stan	South Altai, Katon-Karagai	49.2133364	85.3892447	This study	2021	H47	OR837127 (CR), OR837134 (Cyt *b*)	
8	AKML 1	IZK	Kazakh-stan	Kokshetau pine forests, Akmola region, Northern Kazakhstan	53.3331737	69.6829391	This study	2021	H36	OR837128 (CR), OR837135 (Cyt *b*)	
9	R13	ZM	China	Northeastern, Hulunbuir, Inner Mongolia	47	120	Rueness et al. 2014	1934	H29	EU818855	
10	R30	ZM	Russia	Center, Republic of Sakha (Yakutia)	62.26	117.32	Rueness et al. 2014	1974	H30	EU818854	
11	R33	ZM	Russia	Russian Far East, Amur region	51.59	127.4	Rueness et al. 2014	1973	H31	EU818853	
12	R50	ZM	Russia	Russian Far East, Primorsky Krai	43.6	133.6	Rueness et al. 2014	1949	H32	EU818852	
13	R24	ZM	Russia	Center, Republic of Sakha (Yakutia)	62.26	117.32	Rueness et al. 2014	1968	H33	EU818851	
14	R36	ZM	Russia	Russian Far East, Primorsky Krai	45.3	136.36	Rueness et al. 2014	1936	H34	EU818850	
15	R43	ZM	Russia	Russian Far East, Primorsky Krai	45.55	133.44	Rueness et al. 2014	NA	H35	EU818848	
16	R37	ZM	Russia	Russian Far East, Amur region	53.11	130.3	Rueness et al. 2014	1978	H35	EU818849	
17	R42	ZM	Russia	Russian Far East, Khabarovsk Krai	49.31	139.41	Rueness et al. 2014	1932	H36	EU818842	
18	R31	ZM	Russia	Russian Far East, Primorsky Krai	45.26	137.6	Rueness et al. 2014	1966	H36	EU818843	
19	R59	ZM	Russia	Russian Far East, Kamchatka Krai	52.34	158.23	Rueness et al. 2014	1967	H36	EU818845	
20	R14 1	ZIN	Russia	Russian Far East, Khabarovsk Krai	59.18	143.16	Rueness et al. 2014	1935	H36	EU818847	
21	R75	ZM	Russia	Southwest, Republic of North Ossetia–Alania	43.1	44.42	Rueness et al. 2014	1934	H37	EU818846	
22	R38	ZM	Russia	Russian Far East, Khabarovsk Krai	49.31	139.41	Rueness et al. 2014	1932	H38	EU818844	
23	H18	AFD	Russia	Center, Kirov	59.605	51.28137	Ratkiewicz et	2011	H39	KM000080	

				Republic	451		al. 2014				
24	R57	ZM	Russia	Southwest, Republic of North Ossetia–Alania	43.2	44.35	Ratkiewicz et al. 2014	1911	H1	EU818858	
25	R170	ZIN	China	Center, Qinghai Province	37.3	100	Ratkiewicz et al. 2014	1908	H1	EU818861	
26	SFHN1	AFD	Iran	Isfahan, Fereydunshahr	32.85833333	50.08666667	Behzadi et al., 2022	2016	H1	OM743788	
27	SFHN2	AFD	Iran	Isfahan, Fereydunshahr, Buin Mian Dasht	32.99583333	50.78416667	Behzadi et al., 2022	2016	H1	OM743789	
28	MRKZ2	AFD	Iran	Markazi, Zarandieh	35.48444444	50.00527778	Behzadi et al., 2022	2013	H1	OM743787	
29	GHZV3	AFD	Iran	Ghazvin, Avaj	35.5575	49.18138889	Behzadi et al., 2022	2015	H1	OM743778	
30	THRN3	AFD	Iran	Tehran, Shymranat, Zayegan	35.97027778	51.59944444	Behzadi et al., 2022	2017	H1	OM743781	
31	SMNN1	AFD	Iran	Semnan, Parvar PA	36.00138889	53.47388889	Behzadi et al., 2022	2016	H1	OM743782	
32	THRN1	VAZ	Iran	Tehran	36.00138889	51.51805556	Behzadi et al., 2022	2016	H1	OM743779	
33	THRN2	VAZ	Iran	Tehran	36.00138889	51.51805556	Behzadi et al., 2022	2016	H1	OM743780	
34	MRKZ1	AFD	Iran	Markazi	36.14083333	50.20083333	Behzadi et al., 2022	2013	H1	OM743786	
35	MZND1	AFD	Iran	Mazandaran, Amol, Yoush Baladeh	36.22138889	51.77277778	Behzadi et al., 2022	2011	H1	OM743785	SOUTH
36	SMNN2	live-trapped	Iran	Semnan, Shahroud, Deh Mulla	36.38138889	54.55944444	Behzadi et al., 2022	2016	H1	OM743783	
37	GOLS1	GDE	Iran	Golestan, Gorgan, Gharnabad	36.777	54.666	Behzadi et al., 2022	2015	H1	OM743784	
38	MRKN1	AFD	Iran	Azerbaijan Garbi-Marakan PA	38.96722222	45.31527778	Behzadi et al., 2022	2016	H1	OM743790	
39	TIEN2	IZK	Kazakh-stan	Northern Tien Shan, Ile Alatau	43.117756	76.9305139	This study	2016	H46	OR837123 (CR), OR837130 (Cyt *b*)	
40	R176	ZIN	Tajikis-tan	Gorno-Badakhshan region, Vanj District	38.3	72	Rueness et al. 2014	1932	H25	EU818862	
41	R32	ZM	Russia	Russian Far East, Amur region	50.13	130.14	Rueness et al. 2014	NA	H26	EU818860	
42	R15	ZM	Turkiye	Northeast, Kars Region	40.42	43.35	Rueness et al. 2014	1936	H27	EU818859	
43	R174	ZIN	Tajikis-tan	Gorno-Badakhshan region, Vanj District	38.3	72	Rueness et al. 2014	1913	H28	EU818856	
44	R172	ZIN	Tajikis-tan	Southwestern, Gorno-Badakhshan region	38	74	Rueness et al. 2014	1913	H44	EU818857	
45	R73	ZM	Poland	Southwestern, Białystok County	53	23	Rueness et al. 2014	1951	H10	EU818881	
46	R63	ZM	Poland	Southwestern, Białystok County	53	23	Rueness et al. 2014	1951	H11	EU818880	
47	H10		Poland	Northeastern, Nidzica County	53.508185	20.506629	Ratkiewicz et al. 2014	1992	H11	JQ928877	
48	H11		Poland	Northeastern, Hajnówka County	52.79997	23.673855	Ratkiewicz et al. 2014	1992	H11	JQ928878	
49	R89	ZM	Poland	Southwestern, Białystok County	53	23	Rueness et al. 2014	1948	H12	EU818879	WEST 1
50	R72	ZM	Poland	Southwestern, Białystok County	53	23	Rueness et al. 2014	1948	H13	EU818877	
51	R74	ZM	Poland	Southwestern, Białystok County	53	23	Rueness et al. 2014	1951	H13	EU818878	
52	H13	AFD	Lithua-nia	Northeastern, Utena County	55.68096	24.995799	Ratkiewicz et al. 2014	1992	H13	KM000075	
53	R95	ZM	Russia	West, Moscow	55.9	36.34	Rueness et al.	1976	H3	EU818889	

				region			2014				
54	BA1		Estonia	South, Viljandi County	58.317347	25.67302	Hellborg et al., 2002	1998–1999	H4	AY034815	
55	BA2		Finland	Southwest, South Ostrobothnia region	62.500084	24.025659	Hellborg et al., 2002	1998–1999	H4	AY034815	
56	R97	ZM	Russia	West, Smolenskaya region	54.33	33.11	Rueness et al. 2014	1993	H4	EU818888	
57	H3		Estonia	East, Jõgeva County	58.784413	26.093683	Ratkiewicz et al., 2012	1992	H4	JQ928873	
58	H15	AFD	Lithuania	Center, Kaunas County	55.279976	24.312082	Ratkiewicz et al., 2014	1992	H41	KM000077	
59	H14	AFD	Lithuania	North, Šiauliai County	55.562911	23.572032	Ratkiewicz et al., 2014	1992	H42	KM000076	
60	R187	SZM	Russia	South, Altai Krai	53.26	85.26	Rueness et al. 2014	1976	H5	EU818887	
61	SCA		Norway	South, Oppland fylke	60.769343	10.086257	Hellborg et al., 2002	1996-1998	H6	AY034816	
62	SCA		Sweden	North, Västernorrland County	63.610873	17.304869	Hellborg et al., 2002	1996-1998	H6	AY034816	
63	R100	ZM	Russia	West, Perm Krai	58.23	56.48	Rueness et al. 2014	1977	H6	EU818884	
64	R53	ZM	Russia	Center, Sverdlovsk region	59.9	61.52	Rueness et al. 2014	1940	H6	EU818886	
65	H4Cro2		Slovakia	Mountain (Dinaric, Carpathian)	48.916696	19.296332	Sindičić et al., 2013	1997-2009	H6	JN084447	
66	H4		Poland	Northeastern and Southern	53.638962	20.122186	Ratkiewicz et al., 2012	1992	H6	JQ928874	
67	R82	ZM	Russia	Center, Krasnoyarsk Krai	55.59	92.48	Rueness et al. 2014	1977	H7	EU818885	
68	H17		Russia	Center, The Komi Republic	61.704339	52.626818	Ratkiewicz et al., 2014	1992	H7	KM000079	
69	BA2		Estonia	West, Rapla County	58.984587	24.227534	Hellborg et al., 2002	1998–1999	H8	AY034814	
70	R62	ZM	Poland	Southwestern, Białystok County	53	23	Rueness et al. 2014	1951	H8	EU818876	
71	R128	ZIN	Russia	Southwest, The Republic of Adygea	44.27	40.1	Rueness et al. 2014	1929	H8	EU818883	
72	H2		Poland	Northeastern, Białystok County	53.040952	23.53135	Ratkiewicz et al., 2012	1992	H8	JQ928872	
73	R80	ZM	Russia	West, Moscow region	55.5	39.28	Rueness et al. 2014	1967	H9	EU818882	
74	R84	ZM	Russia	Center, Krasnoyarsk Krai	55.59	92.48	Rueness et al. 2014	1977	H14	EU818875	WEST 2
75	R124	ZIN	Russia	West, Novgorod region	59.13	33.34	Rueness et al. 2014	1961	H15	EU818874	
76	R103	ZIN	Russia	Southwestern, the Republic of Bashkortostan	55.24	55.33	Rueness et al. 2014	1929	H16	EU818873	
77	H9		Estonia	Northern, Harju County	59.280034	25.5095423	Ratkiewicz et al., 2012	1992	H16	JQ928876	
78	R158	ZIN	Russia	West, Leningrad region	59.3	30.26	Rueness et al. 2014	1956	H17	EU818872	
79	R65	ZM	Russia	West, Moscow region	55.34	38.55	Rueness et al. 2014	1940	H18	EU818871	
80	R3	ZM	Uzbekistan	Northwestern, the Republic of Karakalpakstan	44.15	60.24	Rueness et al. 2014	1974	H19	EU818870	
81	H8		Finland	Center, Central Finland region	62.405968	25.397668	Ratkiewicz et al., 2012	1992	H19	JQ928875	
82	BA3		Latvia	Center, Vidzeme region	56.91724	25.640578	Hellborg et al., 2002	1997–1998	H20	AY034813	
83	R104	ZIN	Russia	Southwest	40.1	44.27	Rueness et al. 2014	1929	H20	EU818863	
84	R96	ZM	Russia	West, Leningrad region	59.9	30.9	Rueness et al. 2014	1910	H20	EU818867	
85	R186	SZM	Russia	South, Novosibirsk region	55.11	80.19	Rueness et al. 2014	1959	H20	EU818869	

86	R178	SZM	Russia	Center-South, Altai Republic	51.47	87.13	Rueness et al. 2014	1981	H21	EU818868	
87	R94	ZM	Russia	Center, Perm Krai	60.34	55.58	Rueness et al. 2014	1926	H22	EU818866	
88	R81	ZM	Russia	Center, Tyumen region	60.15	69.21	Rueness et al. 2014	1941	H23	EU818865	
89	R99	ZM	Russia	West, The Republic of Karelia	60.9	32.32	Rueness et al. 2014	1910	H24	EU818864	
90	H16	AFD	Belarus	West, Grodno Region	53.867994	25.885372	Ratkiewicz et al., 2014	1992	H40	KM000078	
91	H12		Latvia	West, Vidzeme region	57.578819	24.916789	Ratkiewicz et al., 2012	1992	H43	JQ928879	
92	H1	ZM	Poland	Northeastern, Hajnówka County	52.79997	23.673855	Ratkiewicz et al., 2012	1992	H20	JQ928871	

Note: ZM = Zoological Museum at Lomonosov Moscow State University (Moscow, Russia), ZIN = Zoological Museum at the Zoological Institute of the Russian Academy of Sciences (St. Petersburg, Russia), SZM = Siberian Zoological Muzeum of the Institute of Systematics and Ecology of Animals (Novosibirsk, Russia), GDE = Gorgan Department of Environment, Taxidermy (Gorgan, Iran), AFD = Animals found dead, VAZ = Vakil Abad Zoo (Mashhad, Iran), IZK = Institute of Zoology (Almaty).

Loadings of the principal components

Supplementary Table S5. Loadings of the principal components for the skull, samples from the Altai and Northern Tien Shan. The specimens differ in ZW (the zygomatic width of the skull). Data are presented for the first three principal components (PC1, PC2, PC3).

Factor coordinates of the variables, based on correlations						
Variable	Skull, logarithmic data ((Lynx-skull-ad-ln))			Skull, Burnaby size-corrected data ((BURNABY-Lynx-skull-ad-sad-ln-sort))		
	PC 1	PC 2	PC 3	PC 1	PC 2	PC 3
TSL	-0.933173	-0.149198	-0.178911	-0.787902	-0.258040	0.355306
CBL	-0.888607	-0.140593	-0.073008	-0.715350	-0.181785	0.179765
MPL	-0.745249	-0.192124	-0.203162	-0.396736	-0.410171	0.244037
OAL	-0.854342	-0.098795	-0.239453	-0.750426	0.140515	0.268415
UTRL	-0.927990	-0.098071	0.038151	0.062975	-0.644242	0.404635
C^1P^3L	-0.563552	-0.277402	-0.012033	0.006037	-0.382920	0.218517
ZW	*-0.919987*	*-0.169992*	*0.143664*	*0.123699*	*-0.489238*	*-0.386186*
POC	-0.162743	-0.407922	0.527030	0.571489	-0.277299	-0.201358
IOC	-0.790626	0.021986	0.249870	0.279918	-0.286722	0.104361
MB	-0.789959	-0.147886	-0.245170	-0.286753	0.147145	-0.400800
NCC	-0.904090	-0.075982	0.136066	0.102139	-0.642265	-0.021747
RWP^2	-0.859920	-0.149139	0.030246	0.256390	-0.162182	-0.004893
BBC	-0.570088	-0.411282	-0.025805	0.306435	-0.002582	-0.477880
RWC^1	-0.897784	-0.130248	0.228897	0.326252	-0.502708	-0.085034
MDW	-0.850973	-0.037405	0.187619	0.386801	-0.126968	-0.168603
ZAL	-0.832330	-0.193426	-0.128642	-0.665659	0.087931	0.039038
P^3P^4L	-0.859716	0.367772	-0.033456	0.466078	0.302053	0.576125
MSL	-0.786466	-0.168483	-0.238998	-0.321281	-0.051579	-0.053541
MSW	-0.316811	-0.098215	-0.582963	-0.627216	0.355758	-0.348202
C^1a	-0.746151	0.464885	0.226158	0.256995	0.097360	0.571025
C^1b	-0.733406	0.362674	0.203479	0.249198	0.091188	0.339380
P^3a	-0.093691	0.221105	-0.379608	0.236693	0.281262	0.041395

P^3b	-0.497105	0.532756	-0.278391	0.188552	0.627394	0.523200
P^4a	-0.561072	0.368813	0.099307	0.573101	0.123967	0.196658
P^4b	-0.673283	0.367070	0.123926	0.515601	0.506355	-0.410333
P^4c	-0.465949	0.622787	-0.062313	0.428670	0.009798	0.473097
P^4d	-0.130224	0.055607	0.708318	0.535057	-0.242653	0.036848

Supplementary Table S6. Loadings of the principal components for the mandible, samples from the Altai and Northern Tien Shan. Data are presented for the first three principal components (PC1, PC2, PC3).

Factor coordinates of the variables, based on correlations (*Lynx*-Mandibula-ad-sad-ln)<br>Include condition: v4=1						
Variable	Mandible, logarithmic data (Mandibula-ad-sad-ln)			Mandible, Burnaby size-corrected data (Mandibula-ad-sad-ln-Burnaby)		
	PC 1	PC 2	PC 3	PC 1	PC 2	PC 3
ML	-0.619645	0.640589	0.136698	-0.465173	0.314912	0.143420
JL	-0.529255	0.528604	0.134458	-0.183297	0.514380	0.462043
MDL	-0.187867	-0.117919	0.430030	-0.075854	0.418866	-0.590713
LJH	0.631769	0.252530	0.441618	-0.768314	-0.174055	-0.370712
TMA	0.270740	-0.095936	0.547769	-0.269317	0.180071	-0.607301
MMA	0.403042	0.430440	-0.009107	-0.320900	-0.388215	0.252848
JHM_1	0.012164	-0.336733	-0.235952	-0.026516	-0.070136	-0.356099
JHP_3	0.060430	-0.341378	-0.299969	0.119472	-0.344980	0.141457
P_3M_1L	-0.224828	0.054885	0.502896	0.226977	0.145548	-0.113506
CCL	-0.478294	0.399934	0.496759	-0.434379	0.723306	-0.288041
CM_1L	-0.566730	0.467224	0.019529	-0.111677	0.598576	0.286604
JWM_1	0.569758	-0.366879	0.005725	0.163493	-0.631919	-0.298468
C_1a	-0.500339	-0.283898	-0.330079	0.403641	0.222423	0.373559
C_1b	-0.281191	-0.130693	0.192615	0.253278	0.157793	-0.073484
P_3a	-0.668179	-0.329672	-0.436872	0.708186	0.275441	0.038123
P_3b	0.156777	0.045229	-0.391698	0.460856	-0.197212	0.396834
P_4a	-0.616784	-0.391211	0.011116	0.687264	0.354669	-0.143494
P_4b	0.049878	0.653728	-0.460369	-0.467354	0.167345	0.423109
M_1a	-0.165339	-0.592108	0.430269	0.572043	0.065774	-0.506752
M_1b	-0.013200	-0.592446	0.279398	0.391283	-0.073711	-0.124369
M_1c	-0.497024	-0.530936	0.089241	0.739681	0.196243	-0.017145

Discriminant Analysis

Supplementary Table S7. Mahalanobis Quadratic Distance Matrix for cranium.

Class	Squared Mahalanobis Distances (BURNABY-Lynx-skull-ad-sad-ln-sort-10)<br>Sigma-restricted parameterization						
	2	3	5	6	7	8	9
2	0.0000	180.8823	81.86151	172.6797	103.4370	137.2024	151.4999
3	180.8823	0.0000	87.55312	159.8432	116.1688	220.3194	56.7928
5	81.8615	87.5531	0.00000	69.8807	21.9684	66.8917	41.9056
6	172.6797	159.8432	69.88067	0.0000	79.1246	81.8215	66.0180
7	103.4370	116.1688	21.96844	79.1246	0.0000	50.0913	57.6232
8	137.2024	220.3194	66.89172	81.8215	50.0913	0.0000	108.6408
9	151.4999	56.7928	41.90563	66.0180	57.6232	108.6408	0.0000

Supplementary Table S8. Confidence of Mahalanobis quadratic distances, skull, n>=2.

Class	Tests of Significance of Squared Mahalanobis Distances (BURNABY-Lynx-skull-ad-sad-ln-sort-10)<br>F tests with 25 and 6. degrees of freedom<br>Sigma-restricted parameterization													
	2	2	3	3	5	5	6	6	7	7	8	8	9	9
2			1.447058	0.341146	0.654892	0.789671	1.381438	0.365586	1.354085	0.376373	1.097619	0.496885	2.181598	0.167834
3	1.447058	0.341146			0.700425	0.756454	1.278745	0.408028	1.520755	0.315967	1.762555	0.247771	0.817816	0.672068
5	0.654892	0.789671	0.700425	0.756454			0.559045	0.857706	0.287587	0.987731	0.535134	0.873775	0.603441	0.826732
6	1.381438	0.365586	1.278745	0.408028	0.559045	0.857706			1.035813	0.531640	0.654572	0.789903	0.950659	0.583310
7	1.354085	0.376373	1.520755	0.315967	0.287587	0.987731	1.035813	0.531640			0.655740	0.789054	2.765912	0.103789
8	1.097619	0.496885	1.762555	0.247771	0.535134	0.873775	0.654572	0.789903	0.655740	0.789054			1.564428	0.302098
9	2.181598	0.167834	0.817816	0.672068	0.603441	0.826732	0.950659	0.583310	2.765912	0.103789	1.564428	0.302098		

Supplementary Table S9. Mahalanobis Quadratic Distance Matrix for mandible.

Class	Squared Mahalanobis Distances (Mandibula-ad-sad-ln-Burnaby) Sigma-restricted parameterization						
	2	3	5	6	7	9	11
2	0.00000	85.7032	82.4304	94.5918	32.56822	56.15526	50.71819
3	85.70324	0.0000	106.2082	98.6758	51.53686	55.22478	54.63071
5	82.43039	106.2082	0.0000	132.5325	57.31984	50.04527	73.31399
6	94.59177	98.6758	132.5325	0.0000	77.04298	60.68087	49.51548
7	32.56822	51.5369	57.3198	77.0430	0.00000	12.65524	18.47431
9	56.15526	55.2248	50.0453	60.6809	12.65524	0.00000	10.44194
11	50.71819	54.6307	73.3140	49.5155	18.47431	10.44194	0.00000

Supplementary Table S10. Confidence of Mahalanobis quadratic distances, mandible.

Class	Tests of Significance of Squared Mahalanobis Distances (Mandibula-ad-sad-ln-Burnaby)<br>F tests with 19 and 13. degrees of freedom<br>Sigma-restricted parameterization													
	2	2	3	3	5	5	6	6	7	7	9	9	11	11
2			1.891583	0.121497	1.819346	0.136419	2.087764	0.089173	1.176256	0.389712	2.230956	0.071538	1.343300	0.297206
3	1.891583	0.121497			2.344154	0.060309	2.177903	0.077580	1.861341	0.127519	2.193990	0.075691	1.446925	0.250684
5	1.819346	0.136419	2.344154	0.060309			2.925165	0.026366	2.070203	0.091645	1.988216	0.104221	1.941763	0.112169
6	2.087764	0.089173	2.177903	0.077580	2.925165	0.026366			2.782537	0.032061	2.410751	0.054624	1.311446	0.313107
7	1.176256	0.389712	1.861341	0.127519	2.070203	0.091645	2.782537	0.032061			1.675906	0.172146	0.917442	0.578919
9	2.230956	0.071538	2.193990	0.075691	1.988216	0.104221	2.410751	0.054624	1.675906	0.172146			0.592630	0.854217
11	1.343300	0.297206	1.446925	0.250684	1.941763	0.112169	1.311446	0.313107	0.917442	0.578919	0.592630	0.854217		

Mann–Whitney U-test for adjusted data

Supplementary Table S11. Mann–Whitney U-test for adjusted data of cranium, samples from Altai (7) and Tien Shan (9).

Variable	Mann-Whitney U Test (w/ continuity correction) (BURNABY-Lynx-skull-ad-sad-ln-sort)<br>By variable Region_1<br>Marked tests are significant at p <,05000										
	Rank Sum (7)	Rank Sum (9)	U	Z	*p*-value	*p* Bonferroni correction	Z	*p*-value	Valid N (7)	Valid N (9)	2*1sided
TSL	165.0000	213.0000	42.00000	*1.98023*	*0.047679*	0.001852	*1.98023*	*0.047679*	9	18	*0.046198*
CBL	168.0000	210.0000	39.00000	*2.13453*	*0.032800*	0.001852	*2.13453*	*0.032800*	9	18	*0.030828*
MPL	100.0000	278.0000	55.00000	-1.31158	0.189663	0.001852	-1.31158	0.189663	9	18	0.193573
OAL	159.0000	219.0000	48.00000	1.67162	0.094600	0.001852	1.67162	0.094600	9	18	0.095074
UTRL	104.0000	274.0000	59.00000	-1.10584	0.268796	0.001852	-1.10584	0.268796	9	18	0.274596
C^1P^3L	112.0000	266.0000	67.00000	-0.69437	0.487454	0.001852	-0.69437	0.487454	9	18	0.494770
ZW	62.0000	316.0000	17.00000	*-3.26609*	*0.001091*	*0.001852*	*-3.26609*	*0.001091*	9	18	*0.000466*
POC	103.0000	275.0000	58.00000	-1.15728	0.247161	0.001852	-1.15728	0.247161	9	18	0.252532
IOC	117.0000	261.0000	72.00000	-0.43719	0.661972	0.001852	-0.43719	0.661972	9	18	0.667865
MB	115.0000	263.0000	70.00000	-0.54006	0.589155	0.001852	-0.54006	0.589155	9	18	0.595851
NCC	99.0000	279.0000	54.00000	-1.36301	0.172879	0.001852	-1.36301	0.172879	9	18	0.176278
RWP^2	169.0000	209.0000	38.00000	*2.18596*	*0.028819*	0.001852	*2.18596*	*0.028819*	9	18	*0.026752*
BBC	149.0000	229.0000	58.00000	1.15728	0.247161	0.001852	1.15728	0.247161	9	18	0.252532
RWC^1	113.0000	265.0000	68.00000	-0.64293	0.520270	0.001852	-0.64293	0.520270	9	18	0.527460
MDW	102.0000	276.0000	57.00000	-1.20871	0.226775	0.001852	-1.20871	0.226775	9	18	0.231679
ZAL	170.0000	208.0000	37.00000	*2.23740*	*0.025261*	0.001852	*2.23740*	*0.025261*	9	18	*0.023129*
P^3P^4L	125.0000	253.0000	80.00000	-0.02572	0.979483	0.001852	-0.02572	0.979483	9	18	0.979889
MSL	140.0000	238.0000	67.00000	0.69437	0.487454	0.001852	0.69437	0.487454	9	18	0.494770
MSW	150.0000	228.0000	57.00000	1.20871	0.226775	0.001852	1.20871	0.226775	9	18	0.231679
C^1a	117.0000	261.0000	72.00000	-0.43719	0.661972	0.001852	-0.43719	0.661972	9	18	0.667865
C^1b	124.0000	254.0000	79.00000	-0.07715	0.938503	0.001852	-0.07715	0.938503	9	18	0.939709
P^3a	162.0000	216.0000	45.00000	1.82592	0.067863	0.001852	1.82592	0.067863	9	18	0.067198
P^3b	155.0000	223.0000	52.00000	1.46588	0.142681	0.001852	1.46588	0.142681	9	18	0.145050
P^4a	116.0000	262.0000	71.00000	-0.48863	0.625106	0.001852	-0.48863	0.625106	9	18	0.631433
P^4b	122.0000	256.0000	77.00000	-0.18002	0.857137	0.001852	-0.18002	0.857137	9	18	0.859894
P^4c	111.0000	267.0000	66.00000	-0.74580	0.455789	0.001852	-0.74580	0.455789	9	18	0.463160
P^4d	94.0000	284.0000	49.00000	-1.62019	0.105193	0.001852	-1.62019	0.105193	9	18	0.106105

Supplementary Table S12. Mann–Whitney U-test for adjusted data of mandible, samples from Altai (7) and Tien Shan (9).

Variable	Mann-Whitney U Test (w/ continuity correction) (Mandibula-ad-sad-ln-Burnaby)<br>By variable Region<br>Marked tests are significant at p <,05000										
	Rank Sum (7)	Rank Sum (9)	U	Z	*p*-value	*p* Bonferroni correction	Z	*p*-value	Valid N (7)	Valid N (9)	2*1sided
ML	100.0000	278.0000	55.00000	-1.31158	0.189663	0.002381	-1.31158	0.189663	9	18	0.193573
JL	88.0000	290.0000	43.00000	-1.92879	0.053758	0.002381	-1.92879	0.053758	9	18	0.052511
MDL	113.0000	265.0000	68.00000	-0.64293	0.520270	0.002381	-0.64293	0.520270	9	18	0.527460
LJH	111.0000	267.0000	66.00000	-0.74580	0.455789	0.002381	-0.74580	0.455789	9	18	0.463160
TMA	132.0000	246.0000	75.00000	0.28289	0.777262	0.002381	0.28289	0.777262	9	18	0.781434
MMA	100.0000	278.0000	55.00000	-1.31158	0.189663	0.002381	-1.31158	0.189663	9	18	0.193573
JHM$_1$	173.0000	205.0000	34.00000	*2.39170*	*0.016771*	0.002381	*2.39170*	*0.016771*	9	18	*0.014610*
JHP$_3$	132.0000	246.0000	75.00000	0.28289	0.777262	0.002381	0.28289	0.777262	9	18	0.781434
P$_3$M$_1$L	111.0000	267.0000	66.00000	-0.74580	0.455789	0.002381	-0.74580	0.455789	9	18	0.463160
CCL	95.0000	283.0000	50.00000	-1.56875	0.116707	0.002381	-1.56875	0.116707	9	18	0.118086
CM$_1$L	98.0000	280.0000	53.00000	-1.41445	0.157232	0.002381	-1.41445	0.157232	9	18	0.160110
JWM$_1$	166.0000	212.0000	41.00000	*2.03166*	*0.042189*	0.002381	*2.03166*	*0.042189*	9	18	*0.040507*
C$_1$a	114.0000	264.0000	69.00000	-0.59150	0.554188	0.002381	-0.59150	0.554188	9	18	0.561168
C$_1$b	138.0000	240.0000	69.00000	0.59150	0.554188	0.002381	0.59150	0.554188	9	18	0.561168
P$_3$a	170.0000	208.0000	37.00000	*2.23740*	*0.025261*	0.002381	*2.23740*	*0.025261*	9	18	*0.023129*
P$_3$b	133.0000	245.0000	74.00000	0.33432	0.738135	0.002381	0.33432	0.738135	9	18	0.742944
P$_4$a	170.0000	208.0000	37.00000	*2.23740*	*0.025261*	0.002381	*2.23740*	*0.025261*	9	18	*0.023129*
P$_4$b	127.0000	251.0000	80.00000	0.02572	0.979483	0.002381	0.02572	0.979483	9	18	0.979889
M$_1$a	161.0000	217.0000	46.00000	1.77449	0.075983	0.002381	1.77449	0.075983	9	18	0.075666
M$_1$b	116.0000	262.0000	71.00000	-0.48863	0.625106	0.002381	-0.48863	0.625106	9	18	0.631433
M$_1$c	117.0000	261.0000	72.00000	-0.43719	0.661972	0.002381	-0.43719	0.661972	9	18	0.667865
