# Supplementary figures and images for: Insights into subspecies classification and conservation priorities of Central Asian lynx populations revealed by morphometric and genetic analyses
Source: Sci Rep. 2024 Mar 2;14:5186. doi: 10.1038/s41598-024-55807-x (PMC10908838; doi:10.1038/s41598-024-55807-x)

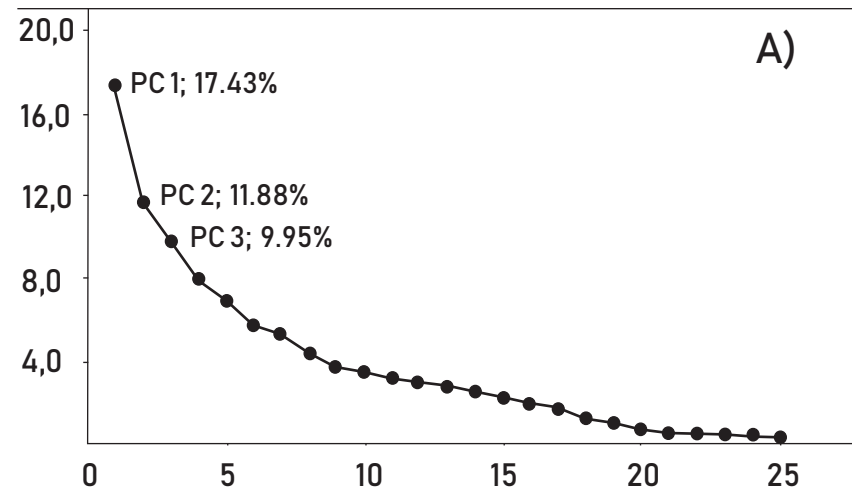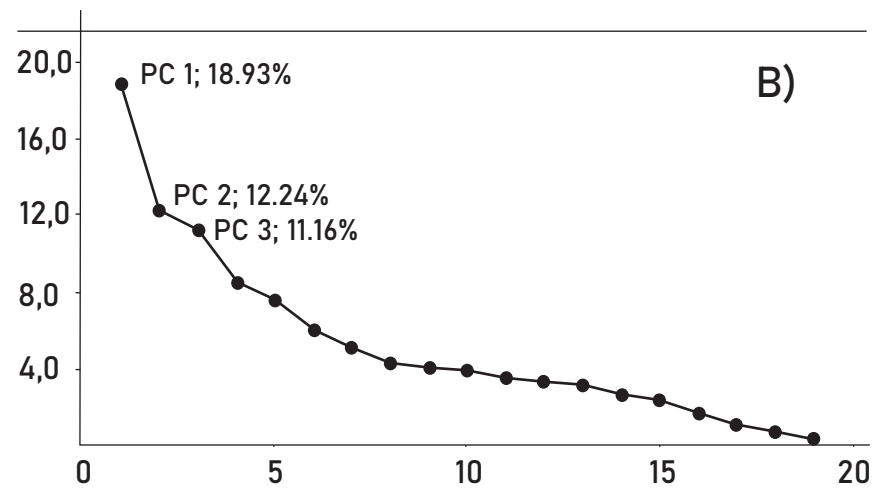

Supplement: Supplementary file 2 — Supplementary Figure S1. [file 41598_2024_55807_MOESM2_ESM.pdf]

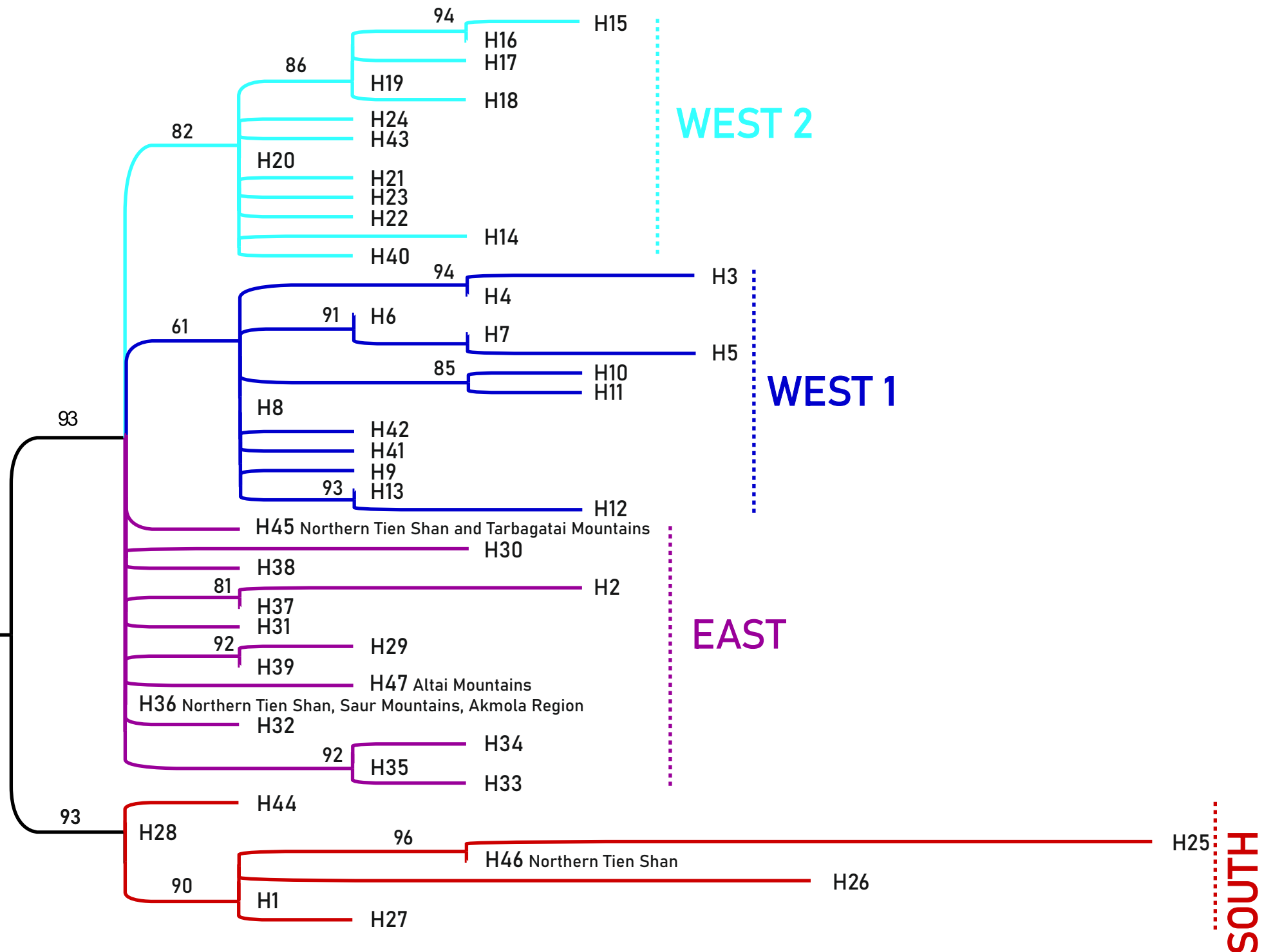

Supplement: Supplementary file 3 — Supplementary Figure S2. [file 41598_2024_55807_MOESM3_ESM.pdf]

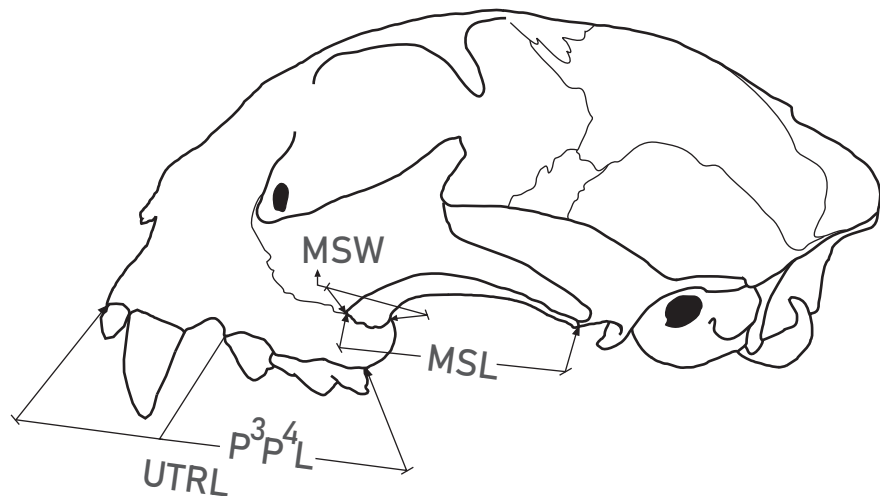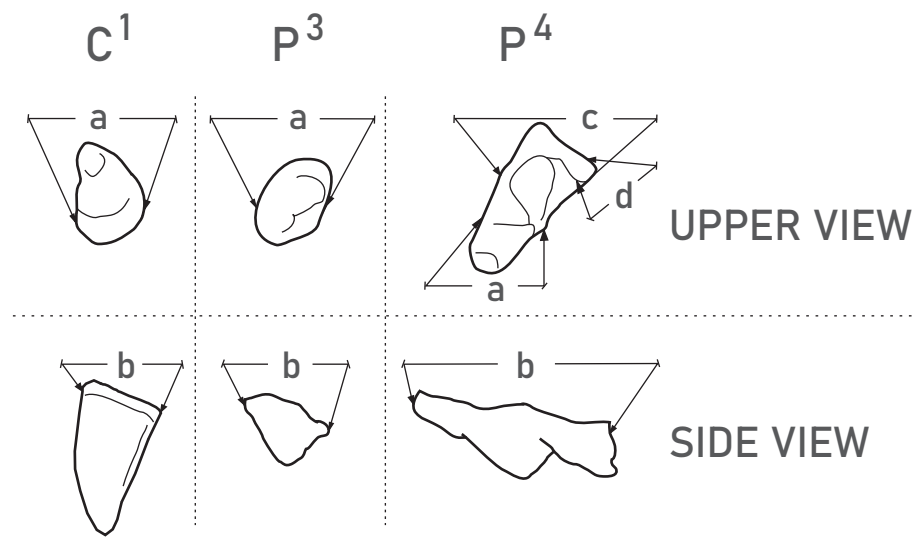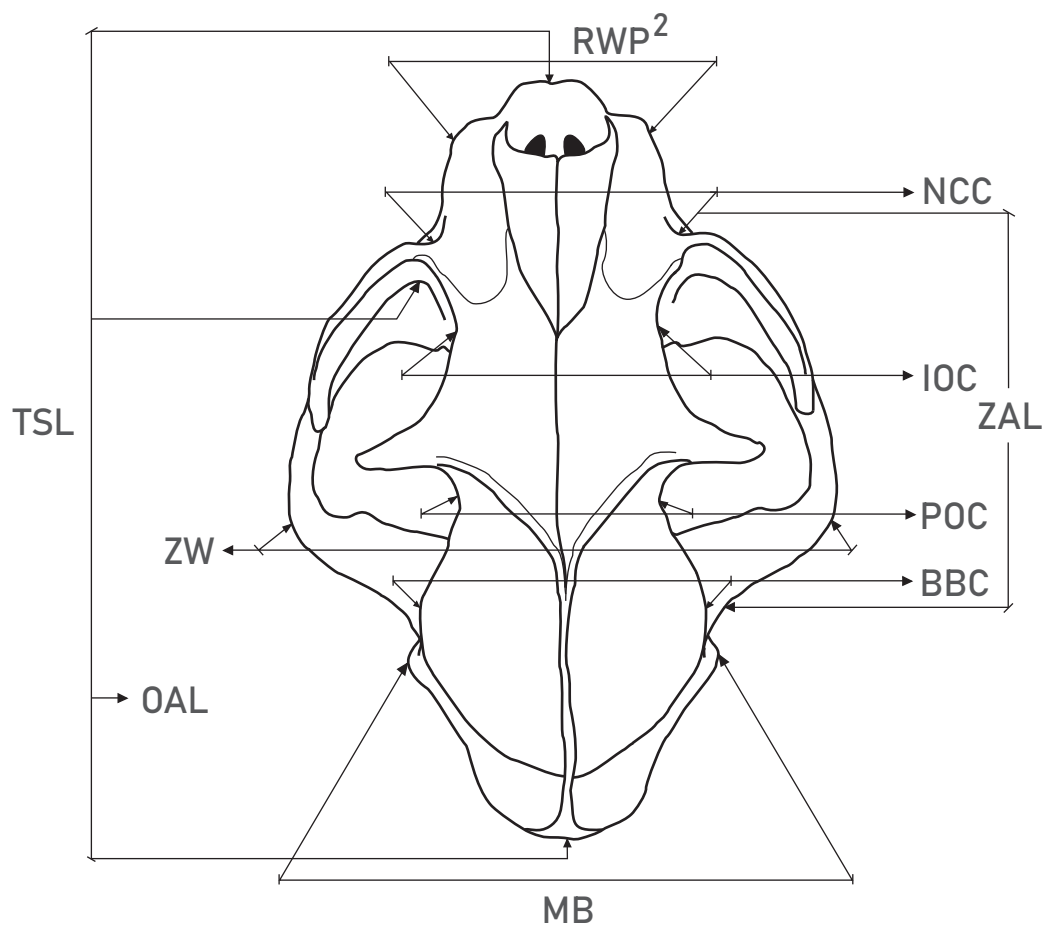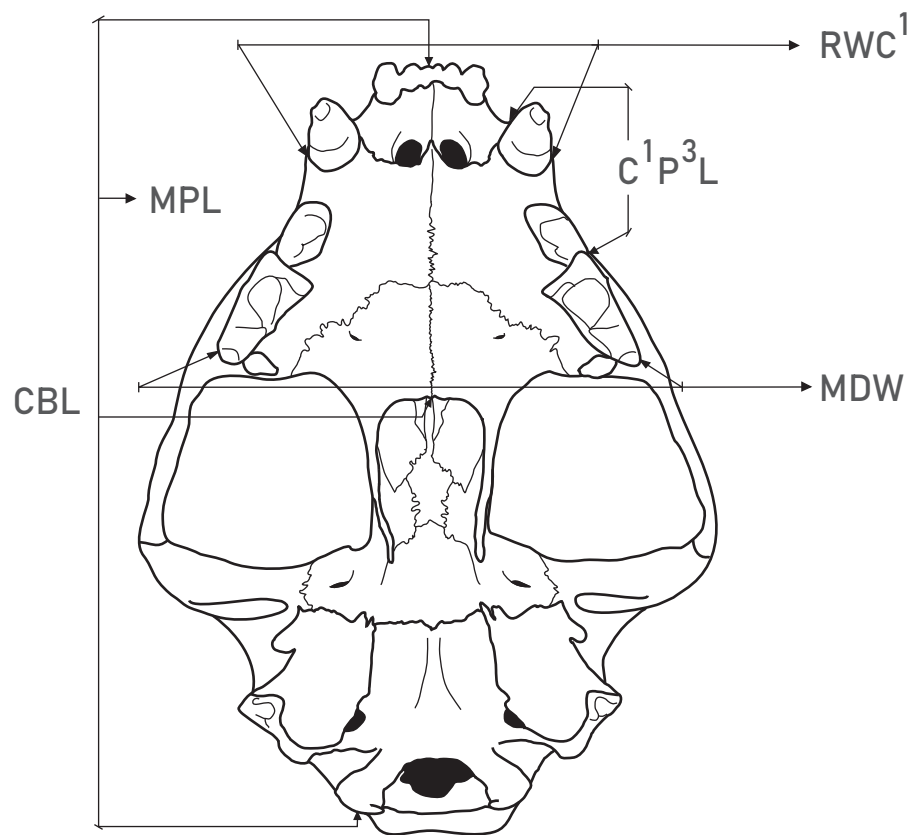

Supplement: Supplementary file 4 — Supplementary Figure S3. [file 41598_2024_55807_MOESM4_ESM.pdf]

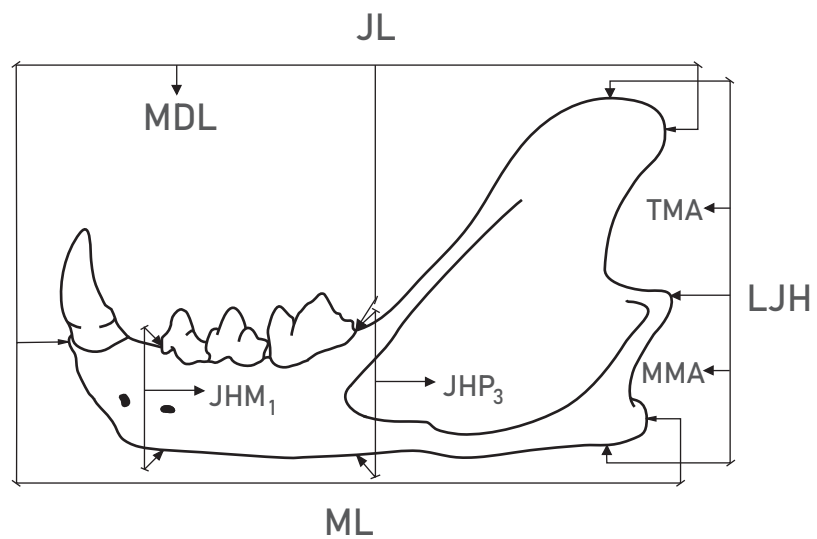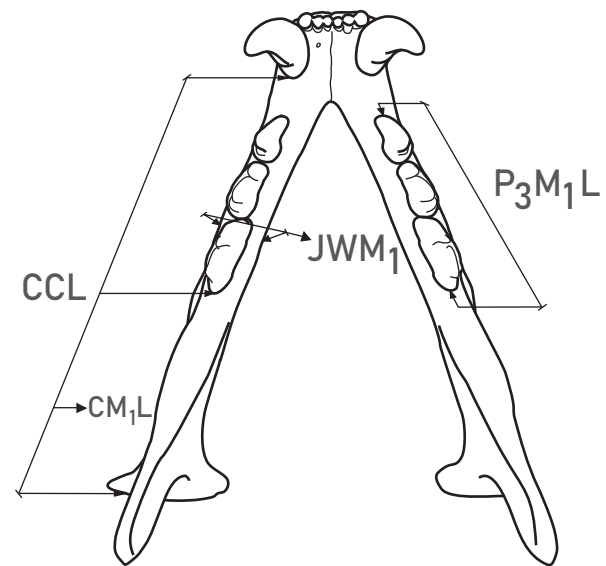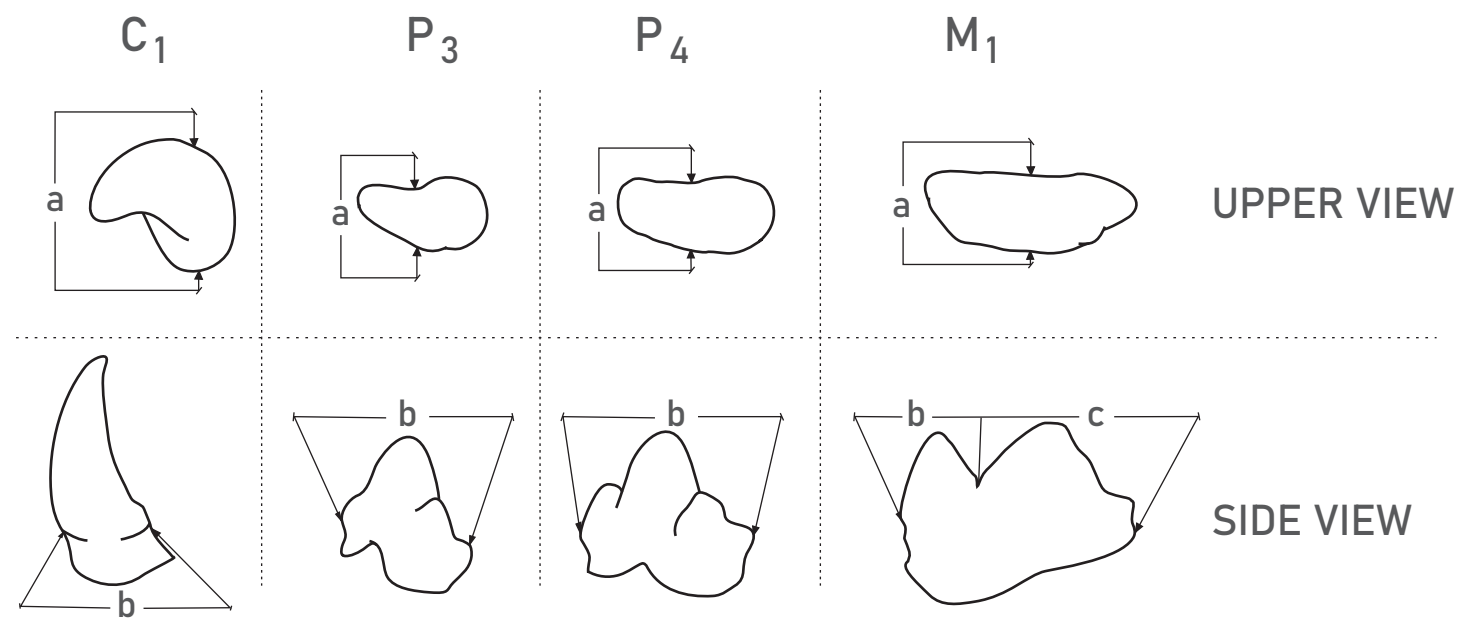

Supplement: Supplementary file 5 — Supplementary Figure S4. [file 41598_2024_55807_MOESM5_ESM.pdf]

$\bar{X} = 153,8$  mm, Ad ♂

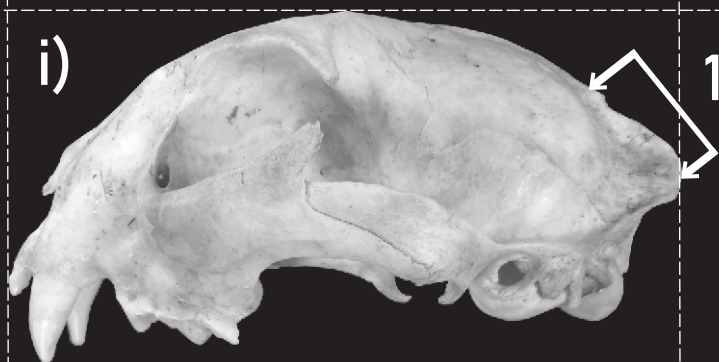

$\bar{X} = 145,6$  mm, Ad ♀

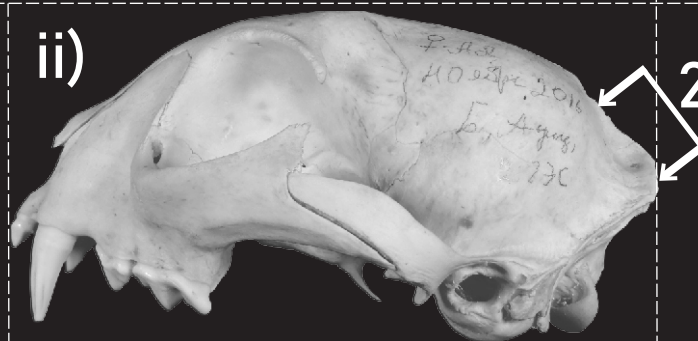

$\bar{X} = 115,1$  mm, Juv

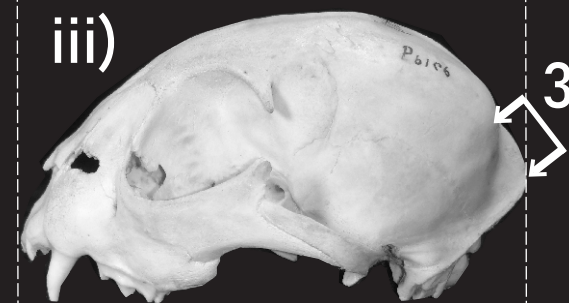

A) Lateral view

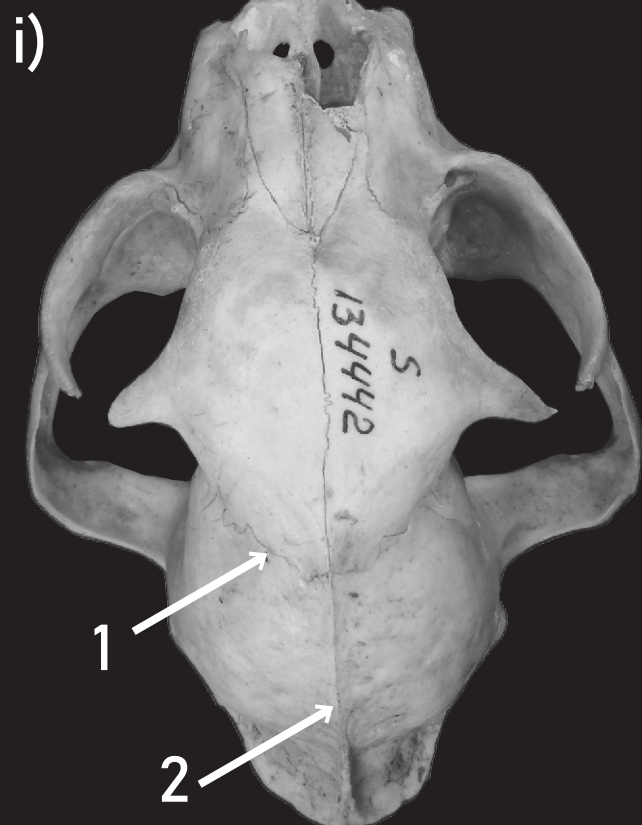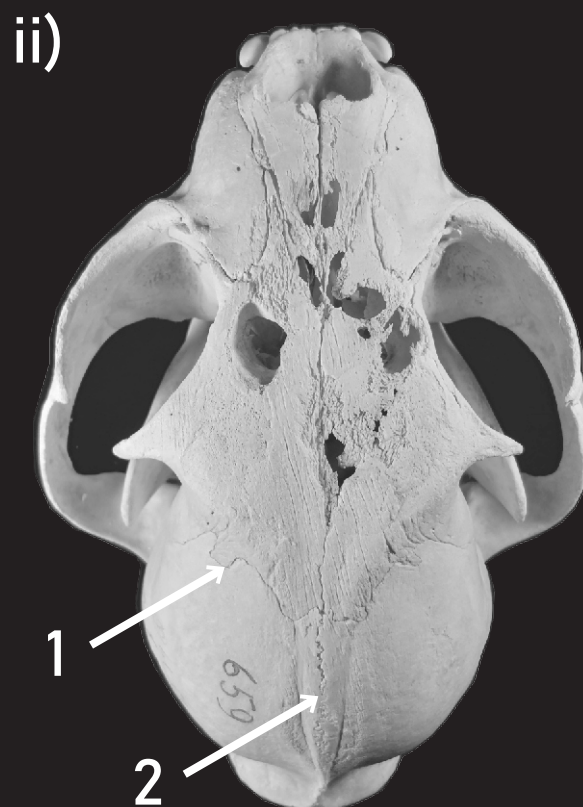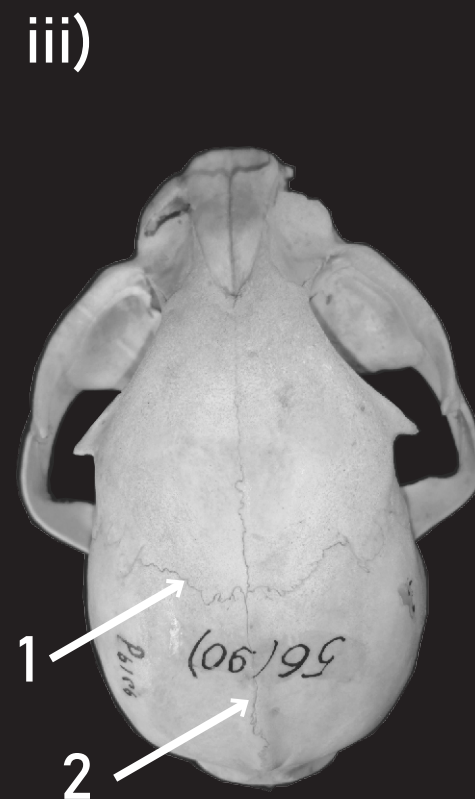

B) Dorsal view

Supplement: Supplementary file 6 — Supplementary Figure S5. [file 41598_2024_55807_MOESM6_ESM.pdf]

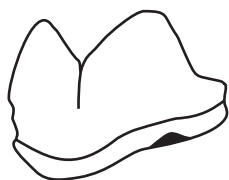

1

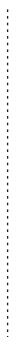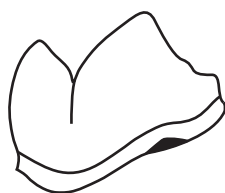

2

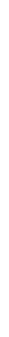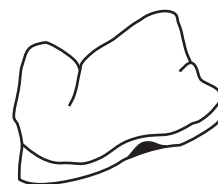

3

Supplement: Supplementary file 7 — Supplementary Figure S6. [file 41598_2024_55807_MOESM7_ESM.pdf]

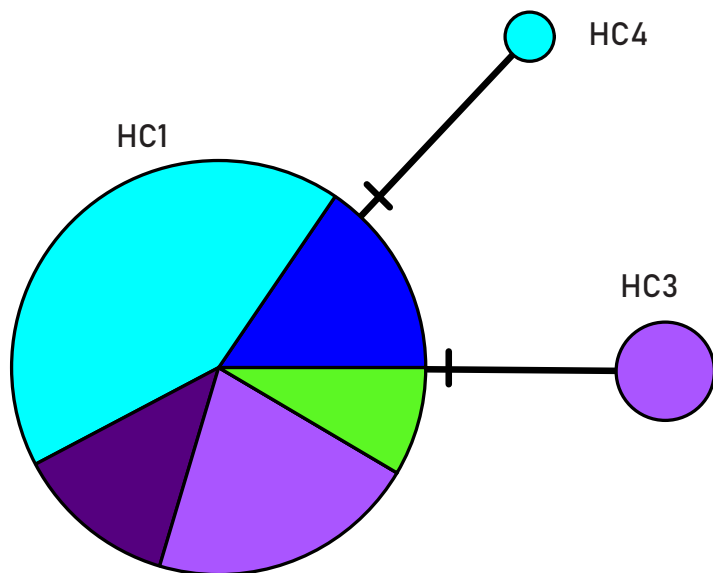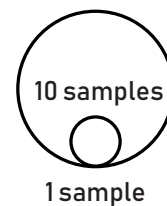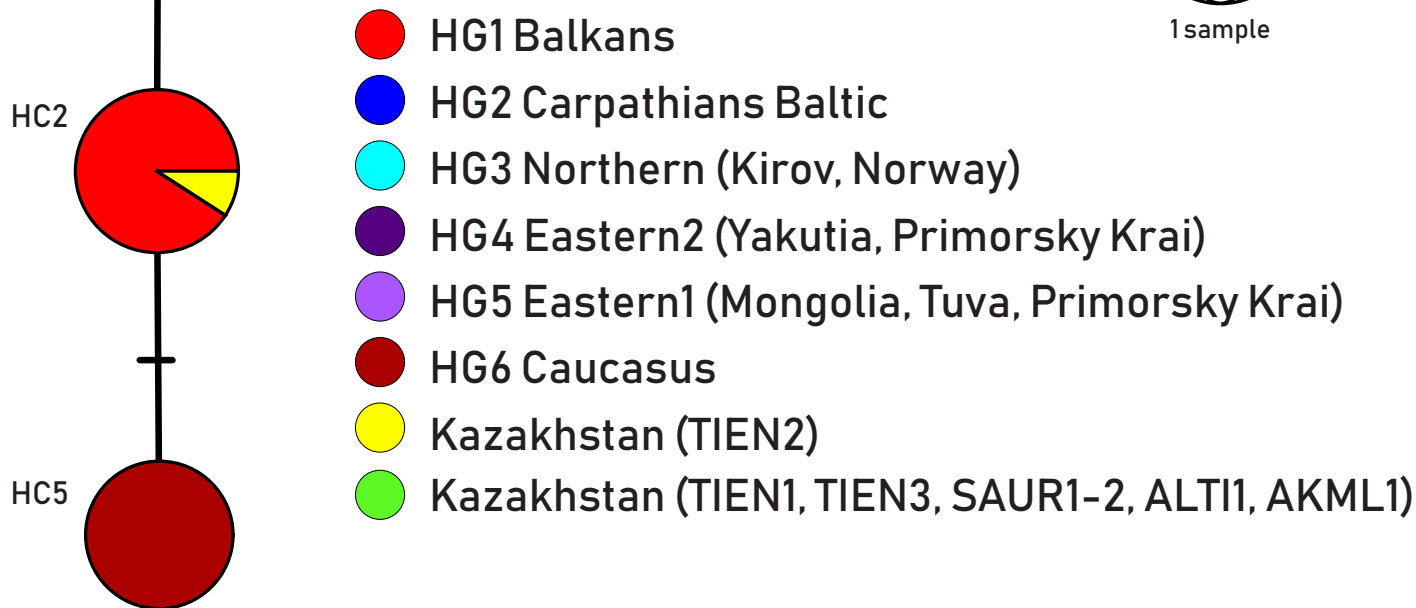

Supplement: Supplementary file 8 — Supplementary Figure S7. [file 41598_2024_55807_MOESM8_ESM.pdf]
